# Supplementary material for: SCRaPL: A Bayesian hierarchical framework for detecting technical associates in single cell multiomics data
Source: PLoS Comput Biol. 2022 Jun 21;18(6):e1010163. doi: 10.1371/journal.pcbi.1010163 (PMC9249169; doi:10.1371/journal.pcbi.1010163)
Supplement: S1 Text — Here we discuss the preprocessing and quality control steps taken in mESC and bESC datasets. This includes aggregating raw epigenetic data from multiple cells, normalizing single cell transcriptomic data, integrating epigenomic and trascriptomic layers and removing low quality data. (PDF) [file pcbi.1010163.s001.pdf]

S1 Text: Data Preprocessing.

SCRaPL: A Bayesian hierarchical framework for detecting technical associates in single cell multiomics data.

Christos Maniatis[1*], Catalina A. Vallejos[2,3*], Guido Sanguinetti[4,1*]

1 School of Informatics, The University of Edinburgh, Edinburgh, UK
2 The Alan Turing Institute, London,UK
3 MRC Human Genetics Unit, Institute of Genetics and Cancer, Western General Hospital, The University of Edinburgh, , Edinburgh, UK
4 International School for Advanced Studies (SISSA-ISA), Trieste, Italy

* s1315538@sms.ed.ac.uk(CM);* catalina.vallejos@ed.ac.uk(CAV);* gsanguin@sissa.it(GS)

Data preprocessing is a vital step prior to inference and is split into two parts, quality control(QC) and integration. Quality control ensures that individual components and joint data satisfy a minimum set of requirements before inference. As single cell sequencing data are stored into different files and formats, appropriate steps need to be considered to bring them in a format understandable by the model. These steps are part of the integration and involve aggregating information for each region in each molecular layer and later joining information between different epigenetic marks using a unique label linked to the region. In this section we give a detailed presentation of integration and quality control steps justifying some of our choices. In many cases integration steps take place between quality controls, so it is natural to present QC and integration steps in the order they appear.

For certain genomic regions when methylation/accessibility data are collected using sc-NOME-seq [1] it is common to have multiple readings. The first step is to average readings for the same genomic region and cell and binarise them in order to get an index for each cytosine. For the binarization step we assume that CpG readings with 50% methylation rate or more are considered methylated. Then using region locations and cell label, methylation and accessibility readings are accumulated into one file. Essentially the script iterates through each cell and aggregates methylated cytosines and total number of cytosines using a window based approach for each region. In this step, user feeds the script with the window length and the minimum coverage a region needs to have in order not to be discarded. Observations in regions with low coverage are marked with 0. These steps take raw binarised data and yield methylation/accessibility or both for genomic region of interest and cell label. For the process described above, we use Bioconductor package BPRMeth [2].

Single cell RNA expression is initially filtered based on the total number of counts per cell, minimum number of expressed genes per cell and distribution of counts. Using scran [3] we then estimate a unique normalization constant for each cell. In this step we take raw expression data, filter low quality cells and estimate per cell normalization constants.

At this point we have aggregated methylation/accessibility data and expression counts with the corresponding normalization constant. For each observation we have a pair of labels indicating genomic region and cell. These labels are used to integrate molecular layers. The final step requires to pass genomic regions through a joint QC control. Regions with zero variance in each component and percentage of expression zeros above 80% are removed. The 80% threshold for expression zeros was set to balance between available non-zero readings in each feature and keeping as many

genomic regions as possible. Since correlation is highly dependent on components variability, it vital to ensure positive variance and avoid inference problems.

For mEBC data much of the preprocessing related to data aggregation has already been done by the publishers. Hence we only map peaks in gene enhancer regions to genes at most 12.5 kbp away. Then we filter features with more than 80% zeros in accessibility or expression.

References

1. Pott S. Simultaneous measurement of chromatin accessibility, DNA methylation, and nucleosome phasing in single cells. Elife. 2017;6:e23203.

2. Kapourani CA, Sanguinetti G. BPRMeth: a flexible Bioconductor package for modelling methylation profiles. Bioinformatics. 2018;34(14):2485-6.

3. Lun AT, McCarthy DJ, Marioni JC. A step-by-step workflow for low-level analysis of single-cell RNA-seq data with Bioconductor. F1000Research. 2016;5.
