# Supplementary material for: SCRaPL: A Bayesian hierarchical framework for detecting technical associates in single cell multiomics data
Source: PLoS Comput Biol. 2022 Jun 21;18(6):e1010163. doi: 10.1371/journal.pcbi.1010163 (PMC9249169; doi:10.1371/journal.pcbi.1010163)
Supplement: S2 Text — In this section we describe step by step the generation of negative control data, explaining our address to problems like missing coverage. (PDF) [file pcbi.1010163.s002.pdf]

S2 Text: Creating negative control datasets.

SCRaPL: A Bayesian hierarchical framework for detecting technical associates in single cell multiomics data.

Christos Maniatis[1*], Catalina A. Vallejos[2,3*], Guido Sanguinetti[4,1*]

1 School of Informatics, The University of Edinburgh, Edinburgh, UK
2 The Alan Turing Institute, London,UK
3 MRC Human Genetics Unit, Institute of Genetics and Cancer, Western General Hospital, The University of Edinburgh, , Edinburgh, UK
4 International School for Advanced Studies (SISSA-ISA), Trieste, Italy

* s1315538@sms.ed.ac.uk(CM);* catalina.vallejos@ed.ac.uk(CAV);* gsanguin@sissa.it(GS)

Negative control datasets were created to understand model's robustness against false positives. The main idea is that if the model mainly detects false positives, then by shattering any correlation structure we expect to detect similar number of features. In this subsection we give a thorough description of the pipeline creating these datasets.

When constructing negative control data the non-missing methylation/accessibility along with expression values are permuted. To keep the analysis simple, we assume that the starting dataset has single cell methylation and expression data. In our analysis we assume that each component is stored in a separate tensor as depicted in Fig AA. Rows and columns of each tensor represent features and cells respectively. Missing methylation are denoted with black boxes. Scrambling cell labels in this setting essentially means that we column-wise permute tensors (without permuting its labels). As there are missing methylation values it is not possible to column-wise permute the entire tensor, because expression values originally paired with a missing methylation will end-up with non-missing ones changing dataset properties and making comparison difficult.

To bypass this issue we iterate through existing features, generating distinct permutation for each molecular layer. For epigentic marks with missing values (ie. methylation and accessibility) we scramble non-missing cells. With expression things are straight-forward as all values are in place but care needs to shown with normalization constants which are tied to specific cells. This is presented in Fig AB. Pseudocode for creating negative control data is presented in Algorithm 1.

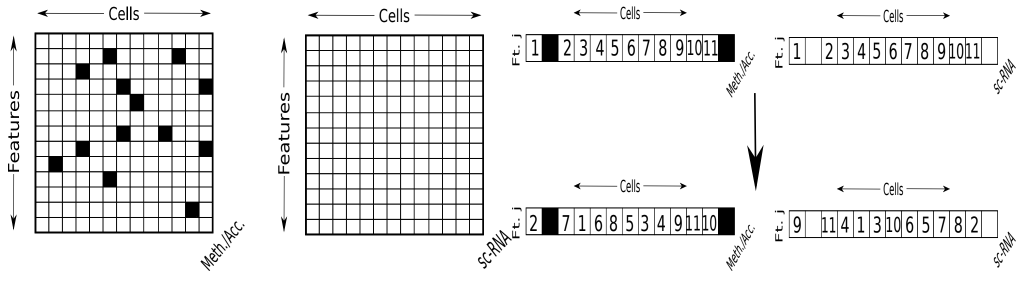

(A) **(B)**

Fig A. (AA) **Sketch of tensors storing methylation/accessibility(left) and expression(right) respectively.** Black boxes represent missing methylation/accessibility data. (AB) Sketch of cell scrambling for a random feature j. It is important to note that despite cells are permuted at the end of scrambling, cell labels remain fixed.

Algorithm 1 Negative control dataset pipeline

Input: Dataset of interest.
Output: Negative control dataset

```
Load dataset of interest.
Compute the number of genomic features J.
for ii = 1, 2, ..., J do
    Compute number of cells I for that genomic region of interest.
    Define 2 vectors with all integers ranging from 1 to I with dimension 1 × I.
    Compute two distinct random permutations for these vectors.
    Apply permutation the permutation in each epigentic mark.
end for
Save updated data into a new file.
```
