# Supplementary material for: SCRaPL: A Bayesian hierarchical framework for detecting technical associates in single cell multiomics data
Source: PLoS Comput Biol. 2022 Jun 21;18(6):e1010163. doi: 10.1371/journal.pcbi.1010163 (PMC9249169; doi:10.1371/journal.pcbi.1010163)
Supplement: S3 Text — We include an extensive analysis of synthetic data experiments used to develop SCRaPL. In particular, we include three sets of experiments that investigate SCRaPL’s performance as a function of cells, zero-inflation and coverage. The first set is performed on data sampled form the model, the second on data sampled from the model and the correlation from a U[−0.8, −0.6], and the third partly sampled from a deep generative model and partly from the model. (PDF) [file pcbi.1010163.s003.pdf]

S3 Text: Synthetic Data.

SCRaPL: A Bayesian hierarchical framework for detecting technical associates in single cell multiomics data.

Christos Maniatis[1*], Catalina A. Vallejos[2,3*], Guido Sanguinetti[4,1*]

1 School of Informatics, The University of Edinburgh, Edinburgh, UK
2 The Alan Turing Institute, London,UK
3 MRC Human Genetics Unit, Institute of Genetics and Cancer, Western General Hospital, The University of Edinburgh, , Edinburgh, UK
4 International School for Advanced Studies (SISSA-ISA), Trieste, Italy

* s1315538@sms.ed.ac.uk(CM);* catalina.vallejos@ed.ac.uk(CAV);* gsanguin@sissa.it(GS)

To assess the estimation performance of SCRaPL, we experimented on synthetic datasets covering scenarios with different numbers of cells and a range of values in terms of methylation coverage, ZI for the expression data along with different latent mean and covariance structures. In this section we focus on estimation accuracy for a series of latent state parameters of potential interest.

We start by considering a situation of perfect model specification (**Experiments 1-3**), in order to assess the identifiability of our model. In this case, we observe that posterior estimates of correlation tend to be unbiased, with an accuracy which increases with the number of cells in the data set (Fig AE). As expected, the performance degrades with increasing levels of ZI (Fig BE). However, we did not observe significant differences across different levels of coverage (Fig CE). Performance for the remaining parameters could be found in Figs A- C. To probe the importance of prior specification, we generated data where the underlying correlation values ρ_j were in an area with low prior mass (**Experiments 4-6**). In this case, we did observe some bias in our estimates (Fig DE), but the latter diminished with increasing sample sizes. Similarly, performance diminishes with increasing ZI levels (Fig EE) and slightly improves upon increasing coverage levels (Fig FE). Performance for the remaining parameters could be found in Figs D- F. As a final test of more severe model mismatch, we evaluated predictive performance in a scenario where we retained the same noise model, but replaced the latent multivariate Gaussian distribution by expression rates inferred using a variational auto-encoder similar to the one described in [scVI; 1] that was trained on the scRNAseq data from [2] (**Experiments 7-9**). Despite the model mismatch, we observed good estimating performance for all latent parameters across a range of simulations (Figs G-I). In all cases, latent means and standard deviations were set as $\mu_{j1} = 4$, $\mu_{j2} = 1$, $\sigma_{j1} = 3$ and $\sigma_{j2} = 2$. Unless otherwise stated, our simulations were based on: $I = 60$ cells, $J = 300$ features, 20% ZI rate on average for the expression data ($\pi_j = 0.20$) and an average methylation coverage (n_{ij}) equal to 275 ($[50, 500]$) across cells and genes.

The first set of data were generated using SCRaPL's generative model. We designed three types of experiments to asses estimation performance as a function of the number of cells, ZI for the expression data and methylation coverage.

- **Experiment 1**: varying numbers of cells ($I \in \{5, 10, 25, 50, 100, 200, 400, 800, 1600\}$) and correlation values sampled from a Beta distribution ($\rho_j \sim \text{Beta}(15, 15)$).
- **Experiment 2**: varying ZI rate ($\pi_j \in \{0.1, 0.2, 0.3, 0.4, 0.5, 0.7, 0.8\}$) and correlation values sampled from a Beta distribution ($\rho_j \sim \text{Beta}(15, 15)$).

- **Experiment 3**: varying methylation coverage (n_{ij} sampled from Uniform distributions with ranges given by $[5, 10]$, $[10, 20]$, $[20, 50]$, $[50, 250]$, $[500, 1000]$) and correlation values sampled from a Beta distribution ($\rho_j \sim \text{Beta}(15, 15)$).

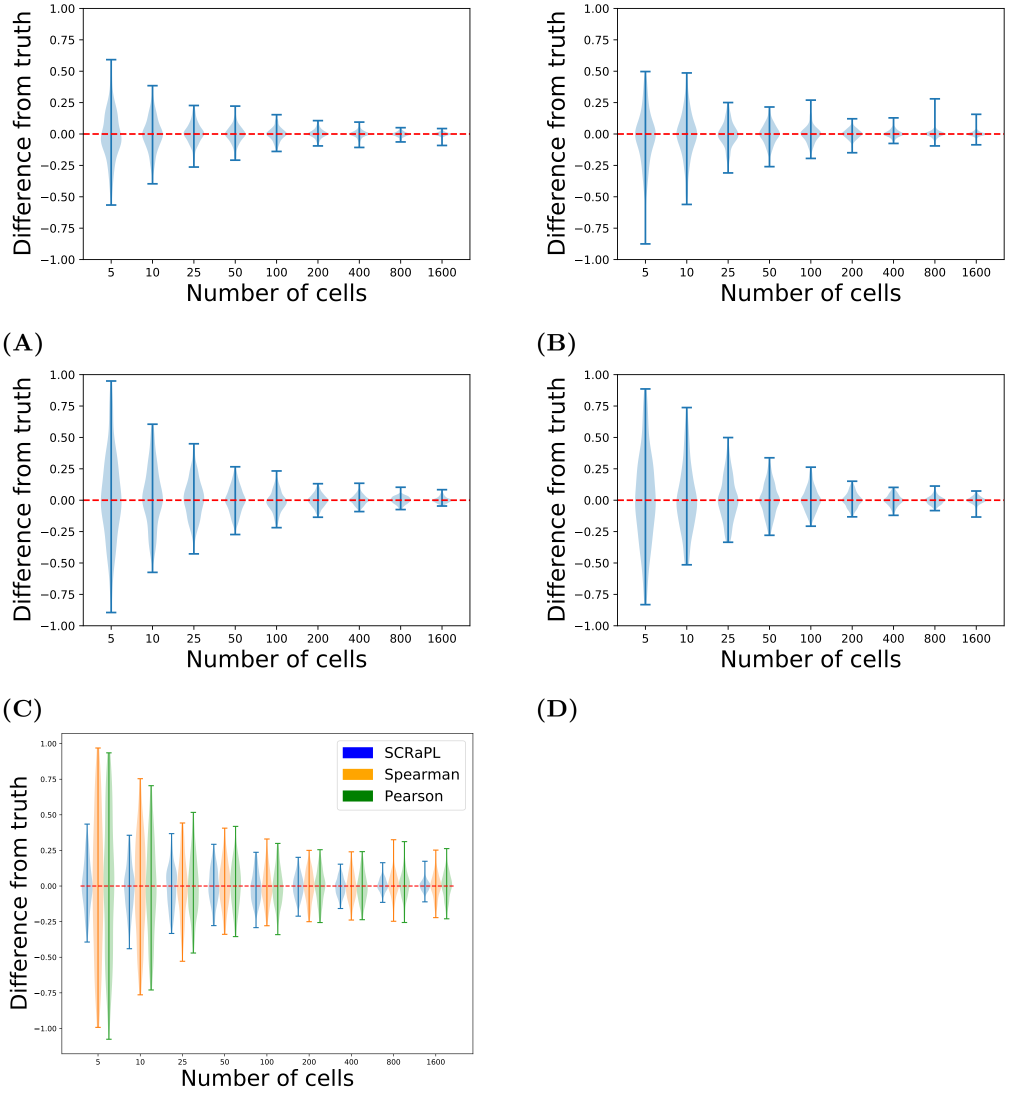

(A) **(B)** **(C)** **(D)** **(E)**

Fig A. Violin plots summarizing difference of posterior mean from data generating parameters for Experiment 1. Differences of posterior estimates from data generating parameters as functions of the number of cells: (AA) methylation mean; (AB) expression mean; (AC) methylation standard deviation; (AD) expression standard deviation; (AE) correlation between expression and methylation.

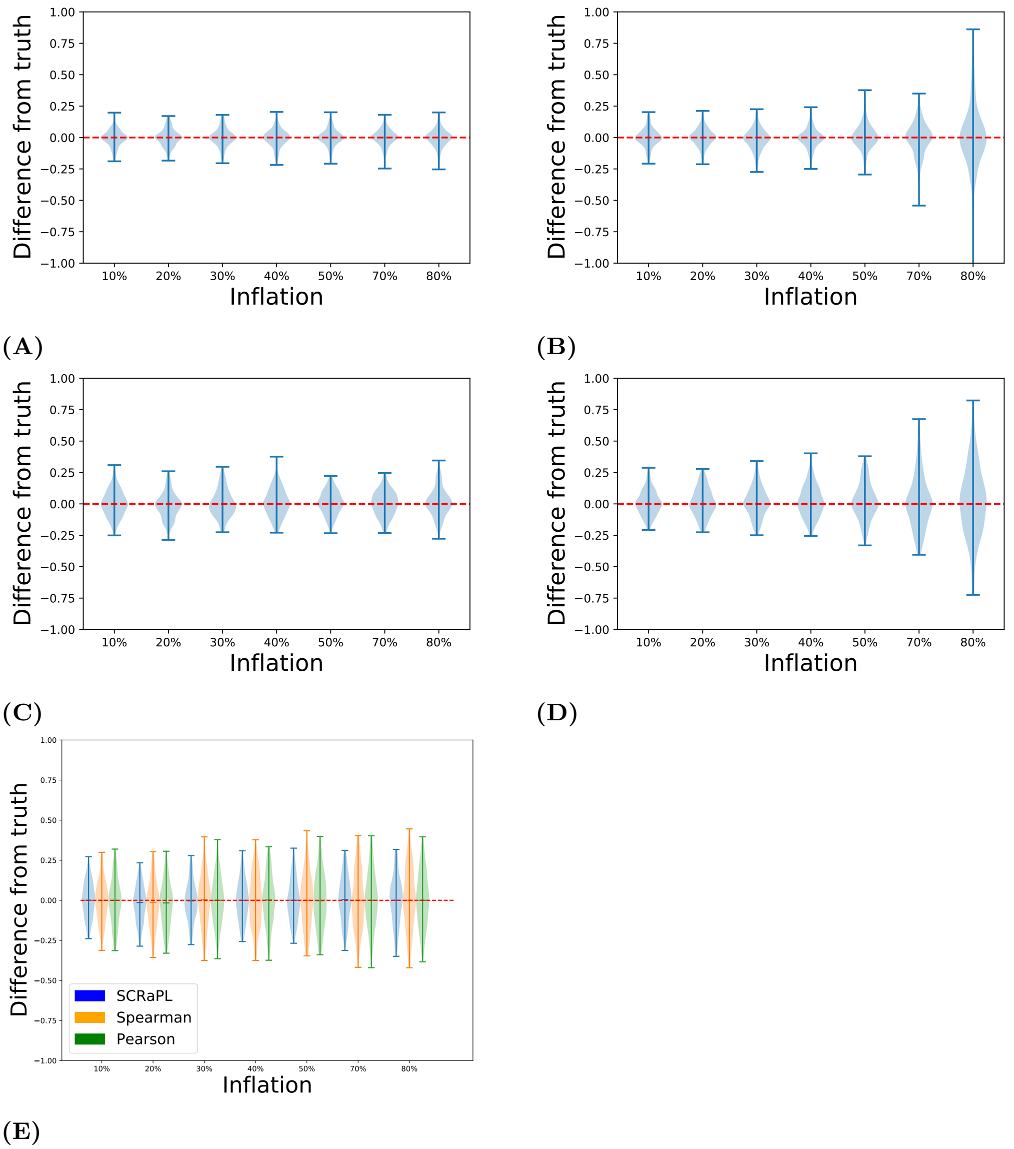

Fig B. Violin plots summarizing difference of posterior mean from data generating parameters for Experiment 2. Differences of posterior estimates from data generating parameters as functions of average expression inflation: (BA) methylation mean; (BB) expression mean; (BC) methylation standard deviation; (BD) expression standard deviation; (BE) correlation between expression and methylation.

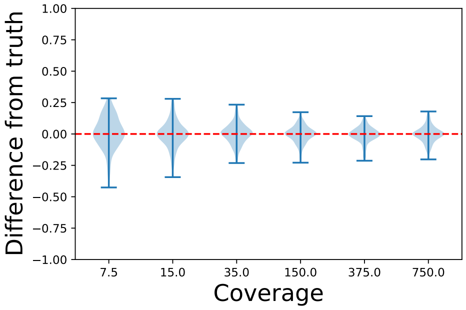

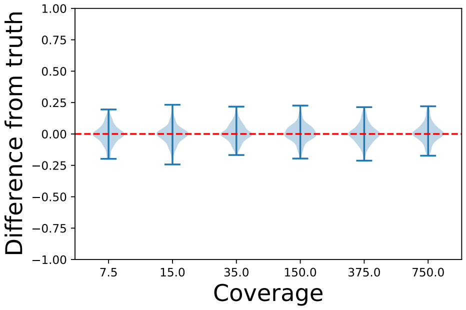

(A) **(B)**

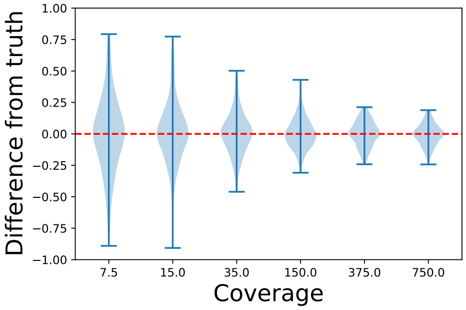

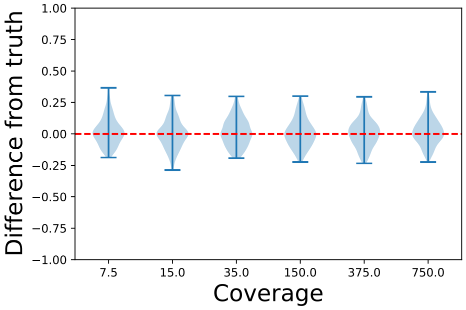

(C) **(D)**

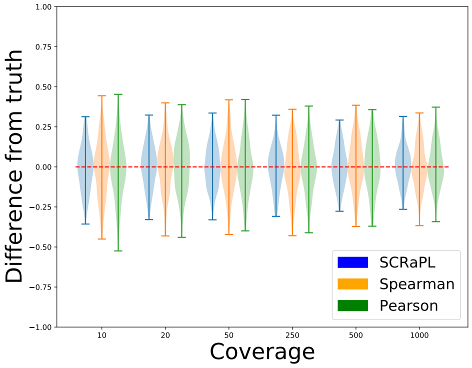

(E)

Fig C. Violin plots summarizing difference of posterior mean from data generating parameters for Experiment 3. Differences of posterior estimates from data generating parameters as functions of average methylation coverage: (CA) methylation mean; (CB) expression mean; (CC) methylation standard deviation; (CD) expression standard deviation; (CE) correlation between expression and methylation.

The second set of data were generated using SCRaPL's generative model where the Beta(15, 15) for correlation ρ_j got replaced by a $U_{[-0.8,-0.6]}$. We designed three types of experiments to asses estimation performance as a function of the number of cells, ZI for the expression data and methylation coverage.

- **Experiment 4**: varying numbers of cells ($I \in \{5, 10, 25, 50, 100, 200, 400, 800, 1600\}$) and correlation values sampled from a Uniform distribution ($\rho_j \sim U_{[-0.8,-0.6]}$).
- **Experiment 5**: varying ZI rate ($\pi_j \in \{0.1, 0.2, 0.3, 0.4, 0.5, 0.7, 0.8\}$) and correlation values sampled from a Uniform distribution ($\rho_j \sim U_{[-0.8,-0.6]}$).

- **Experiment 6**: varying methylation coverage (n_{ij} sampled from Uniform distributions with ranges given by $[5, 10]$, $[10, 20]$, $[20, 50]$, $[50, 250]$, $[500, 1000]$) and correlation values sampled from a Uniform distribution ($\rho_j \sim U_{[-0.8,-0.6]}$).

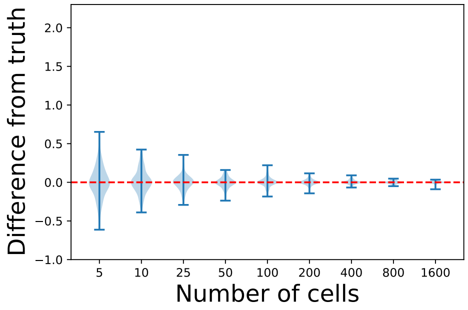

(A)

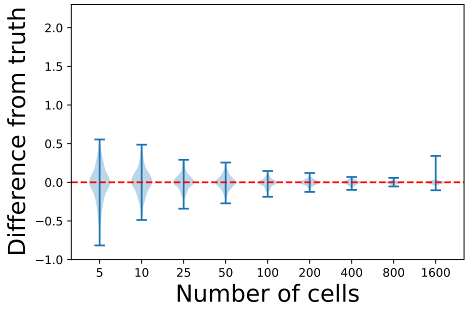

(B)

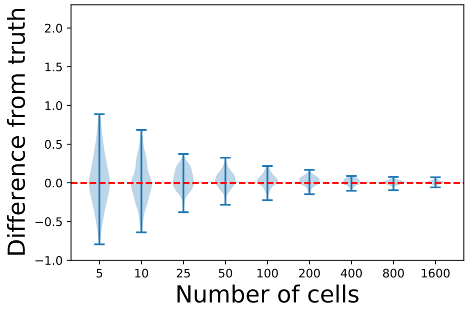

(C)

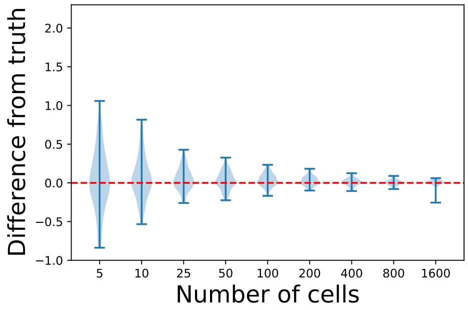

(D)

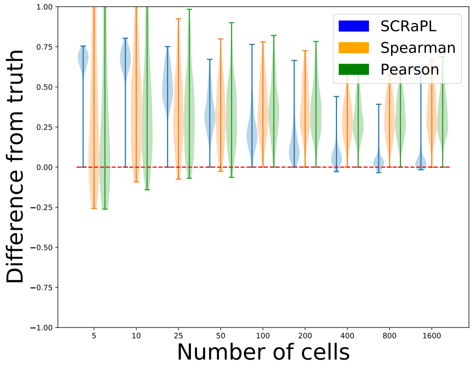

(E)

Fig D. Violin plots summarizing difference of posterior mean from data generating parameters for Experiment 4. Differences of posterior estimates from data generating parameters as functions of the number of cells: (DA) methylation mean; (DB) expression mean; (DC) methylation standard deviation; (DD) expression standard deviation; (DE) correlation between expression and methylation.

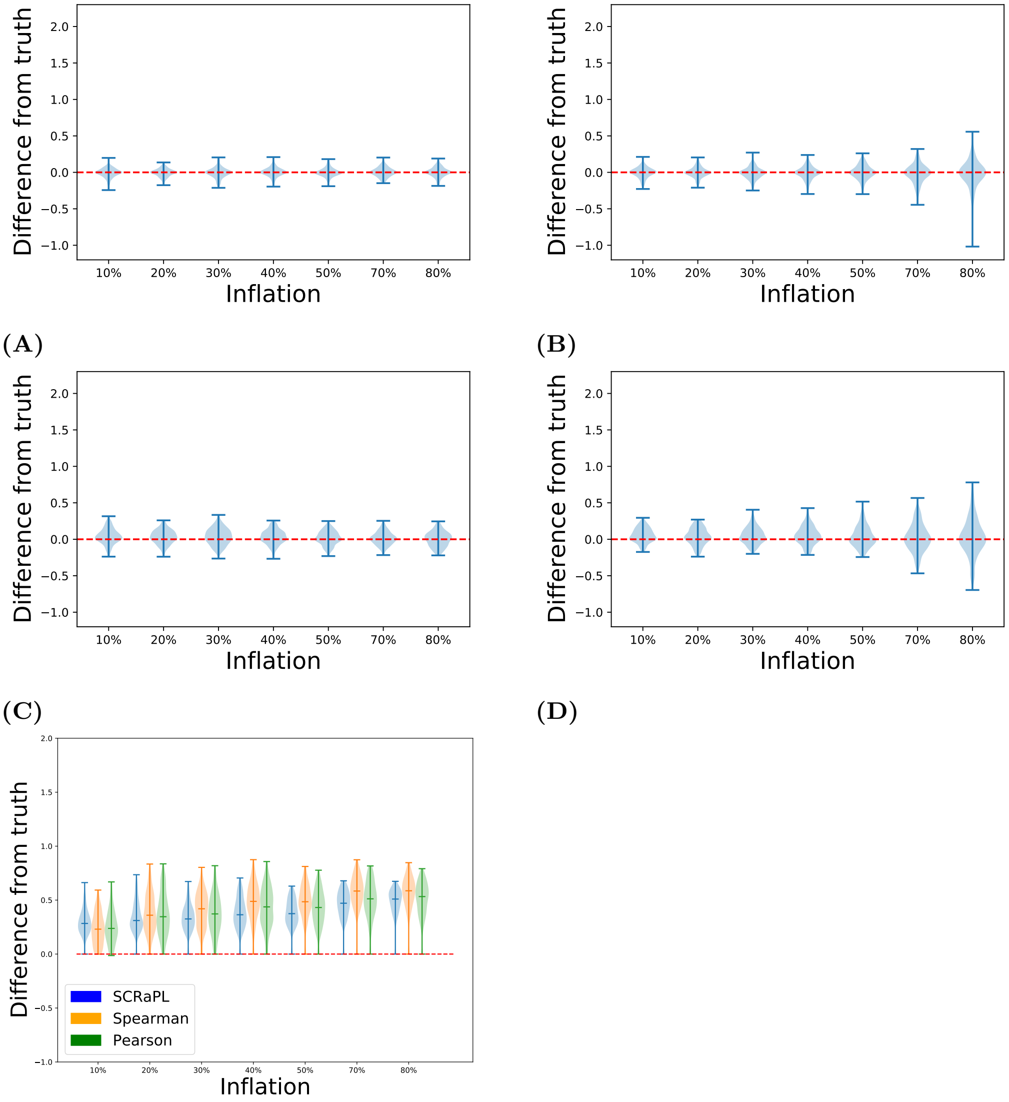

(A) (B) (C) (D) (E)

Fig E. Violin plots summarizing difference of posterior mean from data generating parameters for Experiment 5. Differences of posterior estimates from data generating parameters as functions of average expression inflation: (EA) methylation mean; (EB) expression mean; (EC) methylation standard deviation; (ED) expression standard deviation; (EE) correlation between expression and methylation.

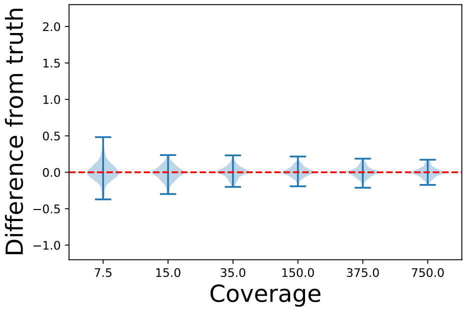

(A)

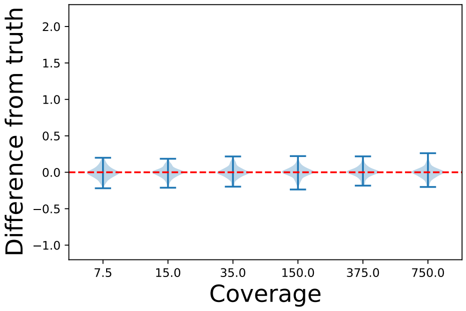

(B)

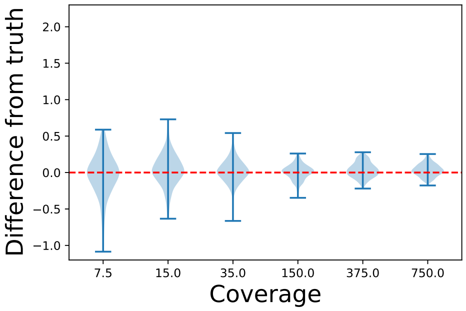

(C)

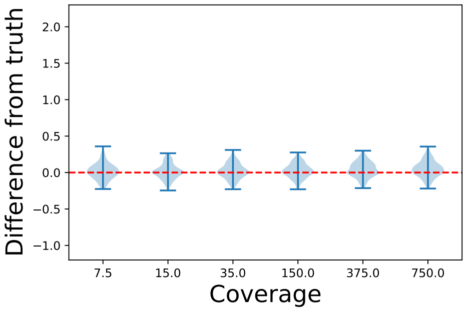

(D)

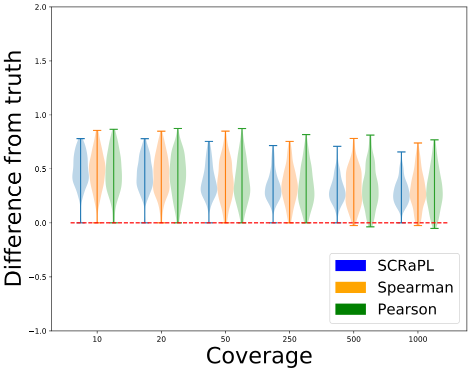

(E)

Fig F. Violin plots summarizing difference of posterior mean from data generating parameters for Experiment 6. Differences of posterior estimates from data generating parameters as functions of average methylation coverage: (FA) methylation mean; (FB) expression mean; (FC) methylation standard deviation; (FD) expression standard deviation; (FE) correlation between expression and methylation.

For the third set of experiments data were partly sampled from a deep generative model similar to the one described in [1] and partly from the model. More precisely the deep generative model was used to generate latent expression and cell specific normalization constants. The rest of the parameters were sampled from the model conditioned on latent expression. We designed three types of experiments to asses estimation performance as a function of the number of cells, ZI for the expression data and methylation coverage.

- **Experiment 7**: varying numbers of cells ($I \in \{5, 10, 25, 50, 100, 200, 400, 800\}$) and correlation values sampled from a Beta distribution ($\rho_j \sim \text{Beta}(15, 15)$).

- **Experiment 8**: varying ZI rate ($\pi_j \in \{0.1, 0.2, 0.3, 0.4, 0.5, 0.7, 0.8\}$) and correlation values sampled from a Beta distribution ($\rho_j \sim \text{Beta}(15, 15)$).
- **Experiment 9**: varying methylation coverage (n_{ij} sampled from Uniform distributions with ranges given by $[5, 10]$, $[10, 20]$, $[20, 50]$, $[50, 250]$, $[500, 1000]$) and correlation values sampled from a Beta distribution ($\rho_j \sim \text{Beta}(15, 15)$).

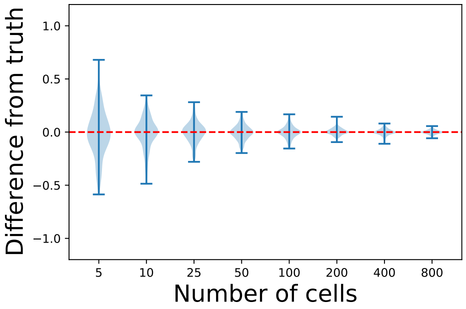

(A)

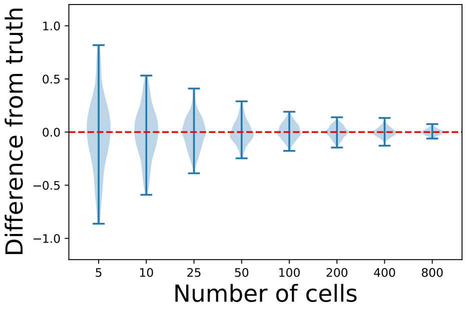

(B)

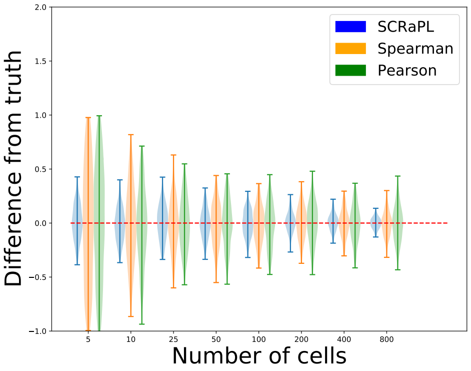

(C)

Fig G. Violin plots summarizing difference of posterior mean from data generating parameters for Experiment 7. Differences of posterior estimates from data generating parameters as functions of the number of cells: (GA) methylation mean; (GB) methylation standard deviation; (GC) correlation between expression and methylation.

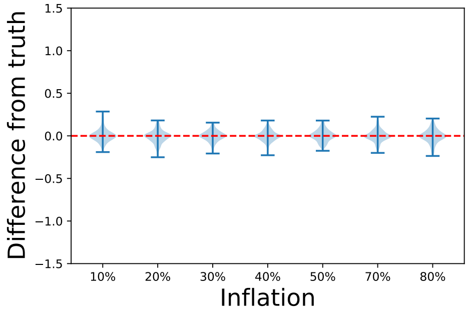

(A)

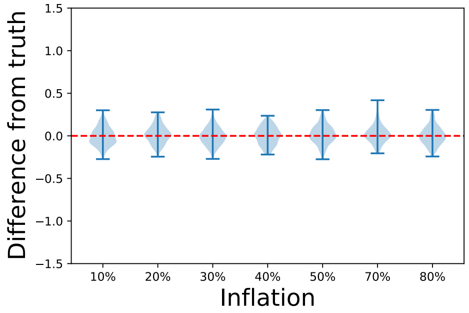

(B)

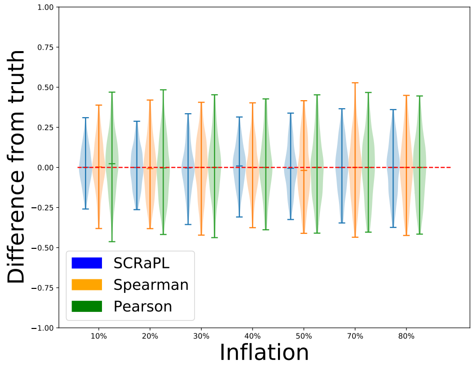

(C)

Fig H. Violin plots summarizing difference of posterior mean from data generating parameters for Experiment 8. Differences of posterior estimates from data generating parameters as functions of average expression inflation: (HA) methylation mean; (HB) methylation standard deviation; (HC) correlation between expression and methylation.

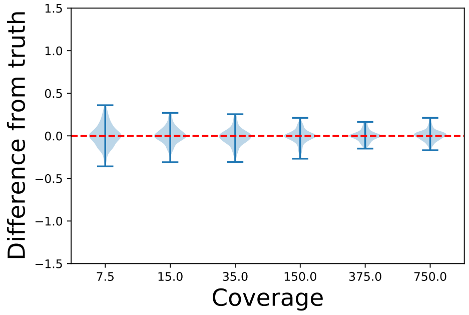

(A)

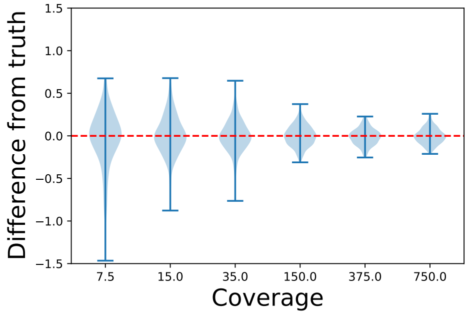

(B)

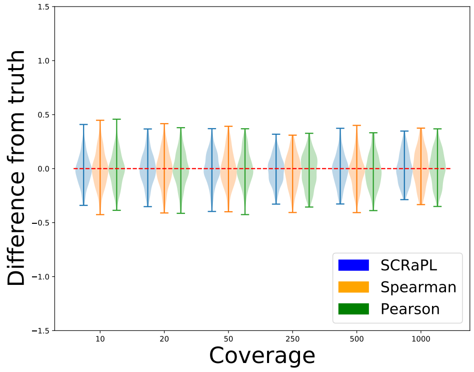

(C)

Fig I. Violin plots summarizing difference of posterior mean from data generating parameters for Experiment 9. Differences of posterior estimates from data generating parameters as functions of average methylation coverage: (FA) methylation mean; (IB) methylation standard deviation; (FE) correlation between expression and methylation.

References

1. Lopez R, Regier J, Cole MB, Jordan MI, Yosef N. Deep generative modeling for single-cell transcriptomics. Nature methods. 2018;15(12):1053-8.

2. Argelaguet R, Clark SJ, Mohammed H, Stapel LC, Krueger C, Kapourani CA, et al. Multi-omics profiling of mouse gastrulation at single-cell resolution. Nature. 2019;576(7787):487-91.
