# Supplementary material for: SCRaPL: A Bayesian hierarchical framework for detecting technical associates in single cell multiomics data
Source: PLoS Comput Biol. 2022 Jun 21;18(6):e1010163. doi: 10.1371/journal.pcbi.1010163 (PMC9249169; doi:10.1371/journal.pcbi.1010163)
Supplement: S4 Text — In this section we lay the detection rate comparisons between SCRaPL and Pearson for methylation/expression of mESC, accessibility/expression of mESC and accessibility/expression of bESC. (PDF) [file pcbi.1010163.s004.pdf]

S4 Text: Negative control experiments.

SCRaPL: A Bayesian hierarchical framework for detecting technical associates in single cell multiomics data.

Christos Maniatis[1*], Catalina A. Vallejos[2,3*], Guido Sanguinetti[4,1*]

1 School of Informatics, The University of Edinburgh, Edinburgh, UK
2 The Alan Turing Institute, London,UK
3 MRC Human Genetics Unit, Institute of Genetics and Cancer, Western General Hospital, The University of Edinburgh, , Edinburgh, UK
4 International School for Advanced Studies (SISSA-ISA), Trieste, Italy

* s1315538@sms.ed.ac.uk(CM);* catalina.vallejos@ed.ac.uk(CAV);* gsanguin@sissa.it(GS)

In this section, we demonstrate that SCRaPL detects significantly more features compared Pearson correlation while keeping false positives below accepted tolerance. To get these results threshold γ was set to be 0.205/0.145/0.115 for mESC, mEBC and synthetic data respectively.

	Orig.	Neg.1	Neg.2	Neg.3	Neg.4	Neg.5
SCRaPL	217	2	2	8	3	0
Pearson	68	0	0	0	0	0
Spearman	85	0	0	0	0	0

Table A. Number of detected features for original and negative control data with SCRaPL,Pearson and Spearman correlation in methylation-expression modalities of mESC data when $\gamma = 0.205$

	Orig.	Neg.1	Neg.2	Neg.3	Neg.4	Neg.5
SCRaPL	4180	31	22	27	10	21
Pearson	816	0	0	0	0	0
Spearman	1	0	0	0	0	0

Table B. Number of detected features for original and negative control data with SCRaPL,Pearson and Spearman correlation in accessibility-expression modalities of mEBC data when $\gamma = 0.145$

	Orig.	Neg.1	Neg.2	Neg.3	Neg.4	Neg.5
SCRaPL	0	0	0	0	0	0
Pearson	0	0	0	0	0	0
Spearman	0	0	0	0	0	0

Table C. Number of detected features for original and negative control data with SCRaPL,Pearson and Spearman correlation in accessibility-expression modalities of mESC cell data when $\gamma = 0.28$

From Tables A/B we see that SCRaPL detects $3 - 5$ times as many features as Pearson correlation, while keeping false positives within accepted tolerance. In accessibility-expression layers of mESC data there does not seem to be enough signal for SCRaPL to yield any useful results.

	Orig.	Neg.1	Neg.2	Neg.3	Neg.4	Neg.5
SCRaPL	715	0	1	4	0	0
Pearson	359	0	0	0	0	0
Spearman	408	0	0	0	0	0

Table D. Number of detected features for original and negative control data with SCRaPL,Pearson and Spearman correlation for synthetic data generated from SCRaPL when $\gamma = 0.115$

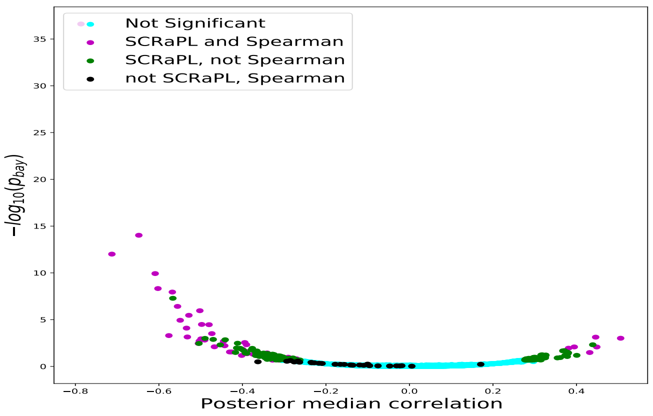

(A)

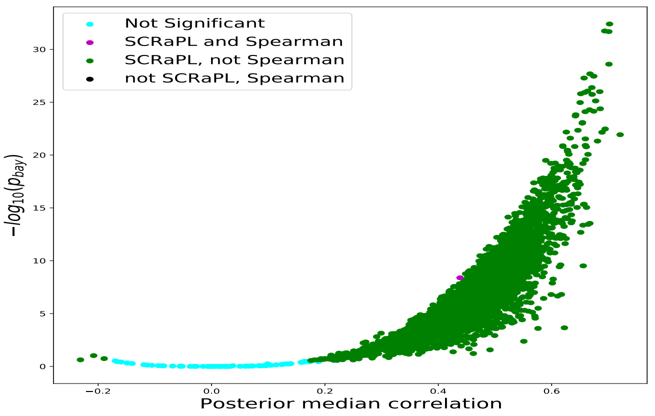

(B)

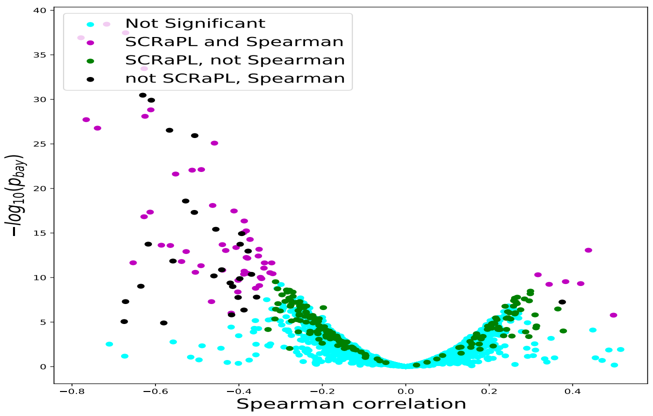

(C)

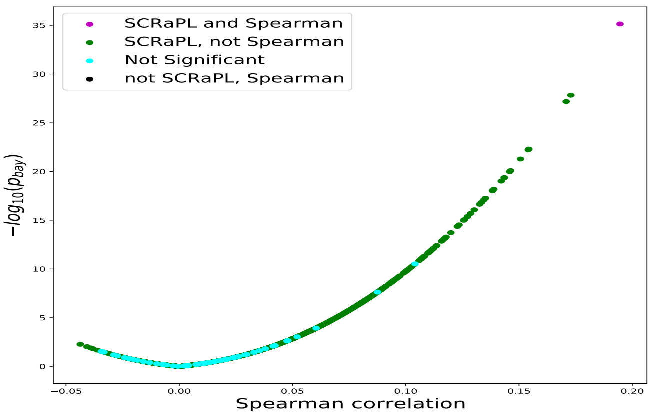

(D)

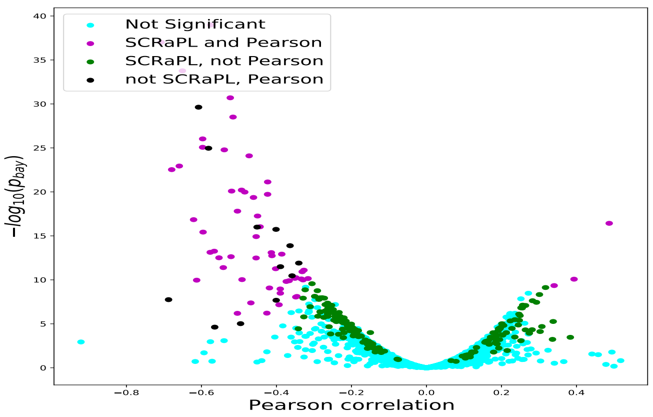

(E)

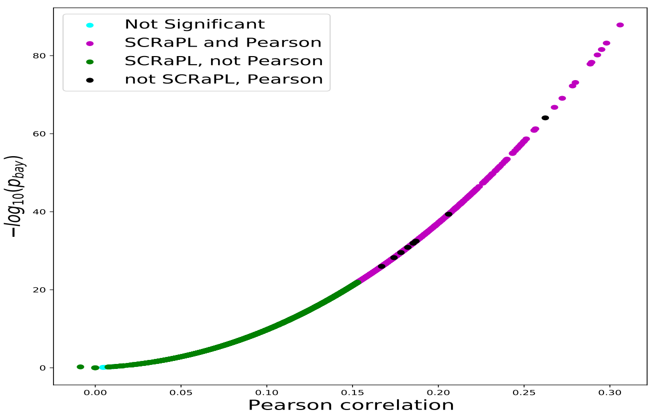

(F)

Fig A. Bayesian and frequentist versions of volcano plots for the mESC and mEBC datasets. Each dot represents a feature and is marked with different color depending the method that labels it as a significant association. (AA) Bayesian volcano plot summarizing significant features as detected by SCRaPL and Spearman correlation in mESC data. (AB) Bayesian volcano plot summarizing significant features as detected by SCRaPL and Spearman correlation in mEBC data. (AC) Frequentist volcano plot summarizing significant features as detected by SCRaPL and Spearman correlation in mESC data. (AD) Frequentist volcano plot summarizing significant features as detected by SCRaPL and Spearman correlation in mEBC data. (AE) Frequentist volcano plot summarizing significant features as detected by SCRaPL and Pearson correlation in mESC data. (AF) Frequentist volcano plot summarizing significant features as detected by SCRaPL and Pearson correlation in mEBC data.
