# Supplementary material for: SCRaPL: A Bayesian hierarchical framework for detecting technical associates in single cell multiomics data
Source: PLoS Comput Biol. 2022 Jun 21;18(6):e1010163. doi: 10.1371/journal.pcbi.1010163 (PMC9249169; doi:10.1371/journal.pcbi.1010163)
Supplement: S5 Text — In this section we present a data driven approach for choosing prior hyper-parameters. (PDF) [file pcbi.1010163.s005.pdf]

S5 Text: Choosing between correlation priors.

SCRaPL: A Bayesian hierarchical framework for detecting technical associates in single cell multiomics data.

Christos Maniatis[1*], Catalina A. Vallejos[2,3*], Guido Sanguinetti[4,1*]

1 School of Informatics, The University of Edinburgh, Edinburgh, UK
2 The Alan Turing Institute, London,UK
3 MRC Human Genetics Unit, Institute of Genetics and Cancer, Western General Hospital, The University of Edinburgh, , Edinburgh, UK
4 International School for Advanced Studies (SISSA-ISA), Trieste, Italy

* s1315538@sms.ed.ac.uk(CM);* catalina.vallejos@ed.ac.uk(CAV);* gsanguin@sissa.it(GS)

While building the model we had to find a systematic way of choosing between beta prior parameters for correlation. Since we did not want to favor neither positive nor negative correlations, we centered the prior at 0. This implies that Beta prior parameters d_1/d_2 were set to be equal. To compare between a strict Beta$(15, 15)$ and a more liberal Beta$(3, 3)$ prior, methylation and expression modalities of mESC data along with a negative control dataset were used. Posterior median correlation as a function of percentage of zeros encountered in each feature for strong and weak priors were compared with Pearson correlation against percentage of zeros. As we can see from Fig A the weak prior tends to be more liberal as the percentage of zeros increases. This behavior is also encountered in the plots when negative control data are used (Fig B), increasing the number of false positives. The strong prior on the other hand both in the true and negative control data yields more conservative results, significantly suppressing false positives.

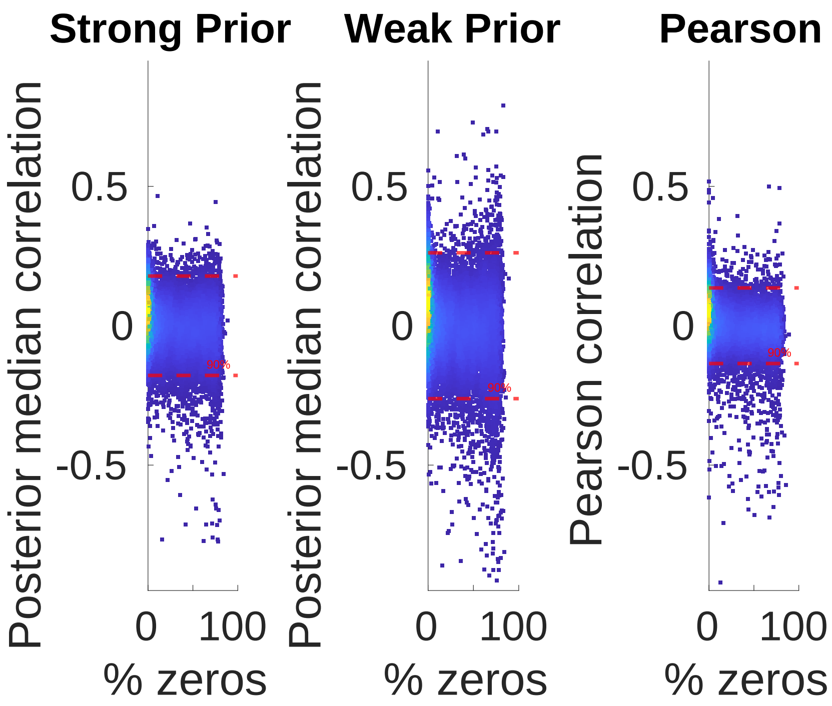

Fig A. Posterior median correlation as function of % of zeros in mESC data for weak prior Beta$(3,3)$ (left), strong prior Beta$(15,15)$ (middle). For comparison we also plot Pearson correlation as a function of % of zeros. Each dot represents a feature.

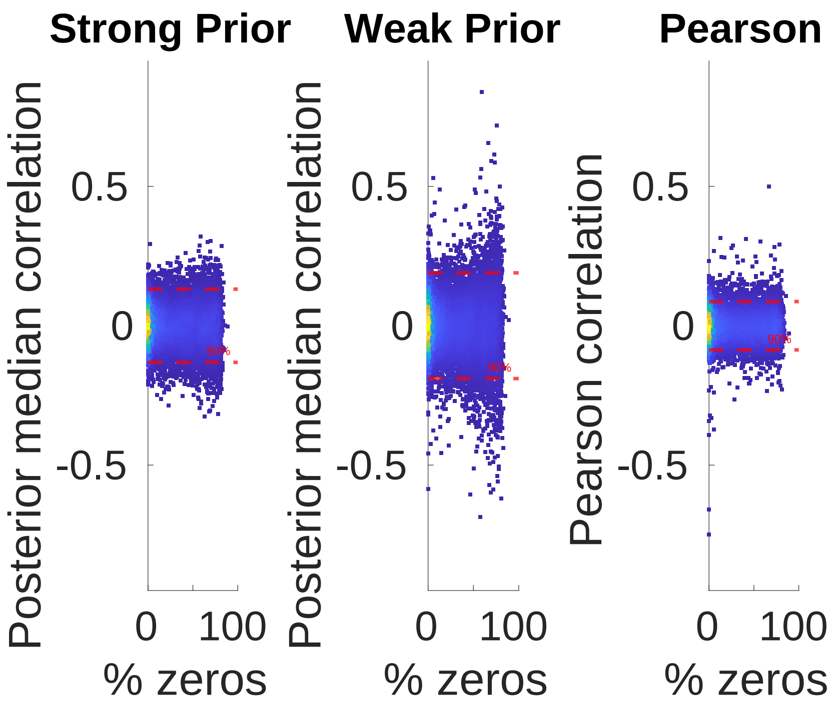

Fig B. Posterior median correlation as function of % of zeros in mESC negative control data for strong prior Beta$(3,3)$ (left), weak prior Beta$(15,15)$ (middle). For comparison we also plot Pearson correlation as a function of % of zeros. Each dot represents a feature.
