# Supplementary material for: SCRaPL: A Bayesian hierarchical framework for detecting technical associates in single cell multiomics data
Source: PLoS Comput Biol. 2022 Jun 21;18(6):e1010163. doi: 10.1371/journal.pcbi.1010163 (PMC9249169; doi:10.1371/journal.pcbi.1010163)
Supplement: S6 Text — In this section a more extended comparison between SCRaPL, Pearson and SPearman predictions is provided. (PDF) [file pcbi.1010163.s006.pdf]

S6 Text: Extended comparisons between SCRaPL, Pearson and Spearman predictions.

SCRaPL: A Bayesian hierarchical framework for detecting technical associates in single cell multiomics data.

Christos Maniatis[1*], Catalina A. Vallejos[2,3*], Guido Sanguinetti[4,1*]

1 School of Informatics, The University of Edinburgh, Edinburgh, UK
2 The Alan Turing Institute, London,UK
3 MRC Human Genetics Unit, Institute of Genetics and Cancer, Western General Hospital, The University of Edinburgh, , Edinburgh, UK
4 International School for Advanced Studies (SISSA-ISA), Trieste, Italy

* s1315538@sms.ed.ac.uk(CM);* catalina.vallejos@ed.ac.uk(CAV);* gsanguin@sissa.it(GS)

In the process of understanding SCRaPL's behavior, we analyzed results in a series of features. We consider the set of associations which are called as significant by at least one method, and split it into 3 categories: agreement between predictions, association labeling as significant by SCRaPL, but not Pearson, and vice-versa. Here we go through some examples omitted from the main text, which demonstrate SCRaPL's superior performance. Since for Spearman correlation there is only only one significant feature, in the relevant subsections we only rely on Pearson for comparison.

1 Classified as significant by SCRaPL and Pearson/Spearman

In this section we present some cases where SCRaPL agrees with Pearson in both mESC and mEBC data. For many of the examples we have good coverage for both methylation and relatively high accessibility/expression. Furthermore in [1] we tend to have observations for a wide range of methylation and expression values.

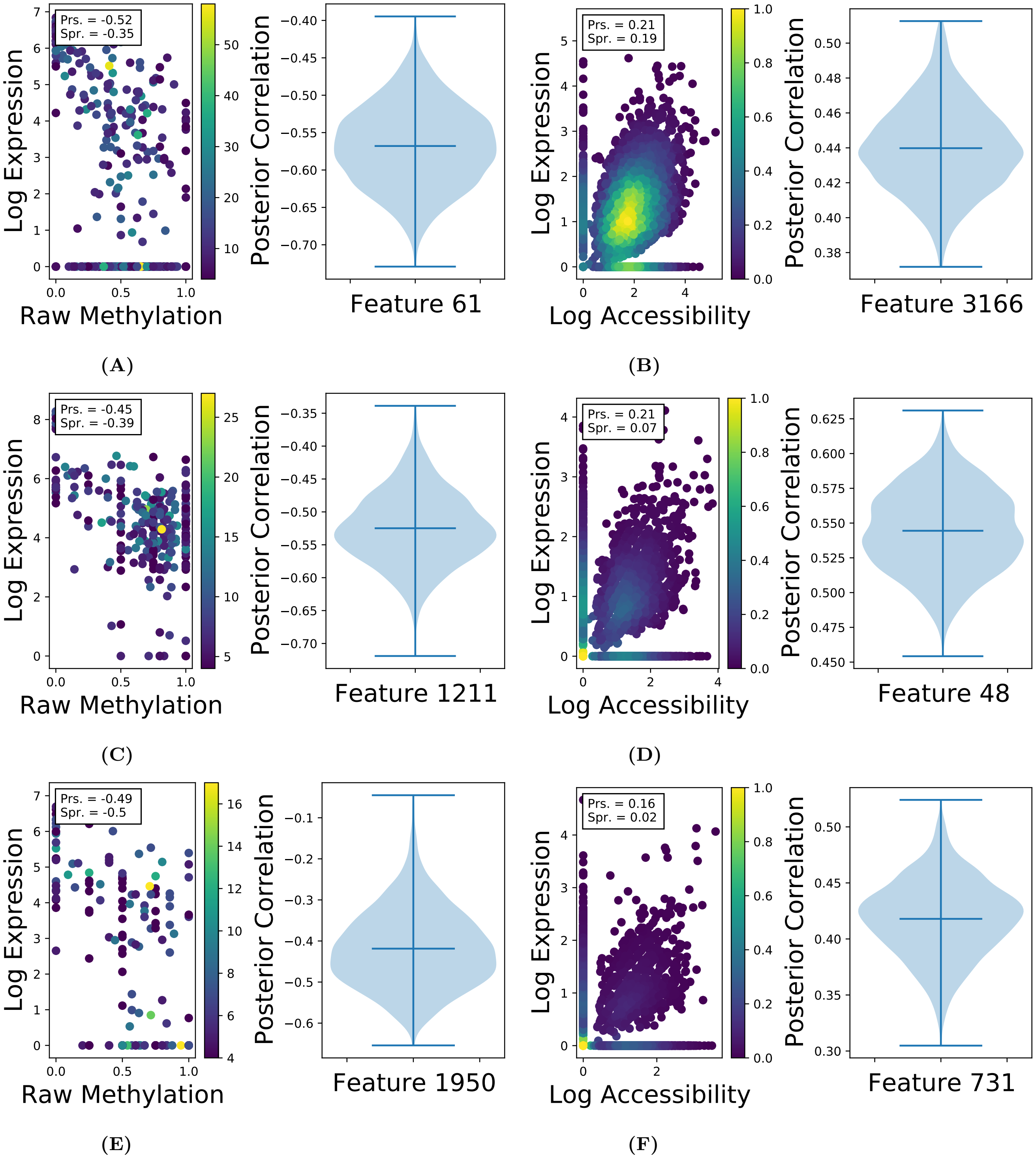

Fig A. Example where both SCRaPL and Pearson identify feature's association as significant in mESC and mEBC datasets. In all figures the left part is a scatter plot of feature's raw data and the right part part is the posterior correlation as inferred by SCRaPL. In features from mESC data (ie. (AA), (AC) and (AE)) each dot of the scatter plot represents a cell reading, color-coded by CpG coverage and the expression axis is in $log(1 + x)$ scale. In features from mEBC data (ie. (AB), (AD) and (AF)) each dot of the scatter plot represents a cell reading, color-coded by space occupation and the accessibility/expression axis are in $log(1 + x)$ scale.

2 Classified as insignificant by SCRaPL and significant by Pearson

In this subsection we generally have features with observations leading to spurious correlations. Some of these problems encountered in observations include, low expression/ number of observations, extremely low coverage and large number of zeros. For many of these features we see that are large areas where we do not have any observation for both methylation and expression. In mEBC data we see an imbalanced ratio of readings with zero accessibility and non-zero expression.

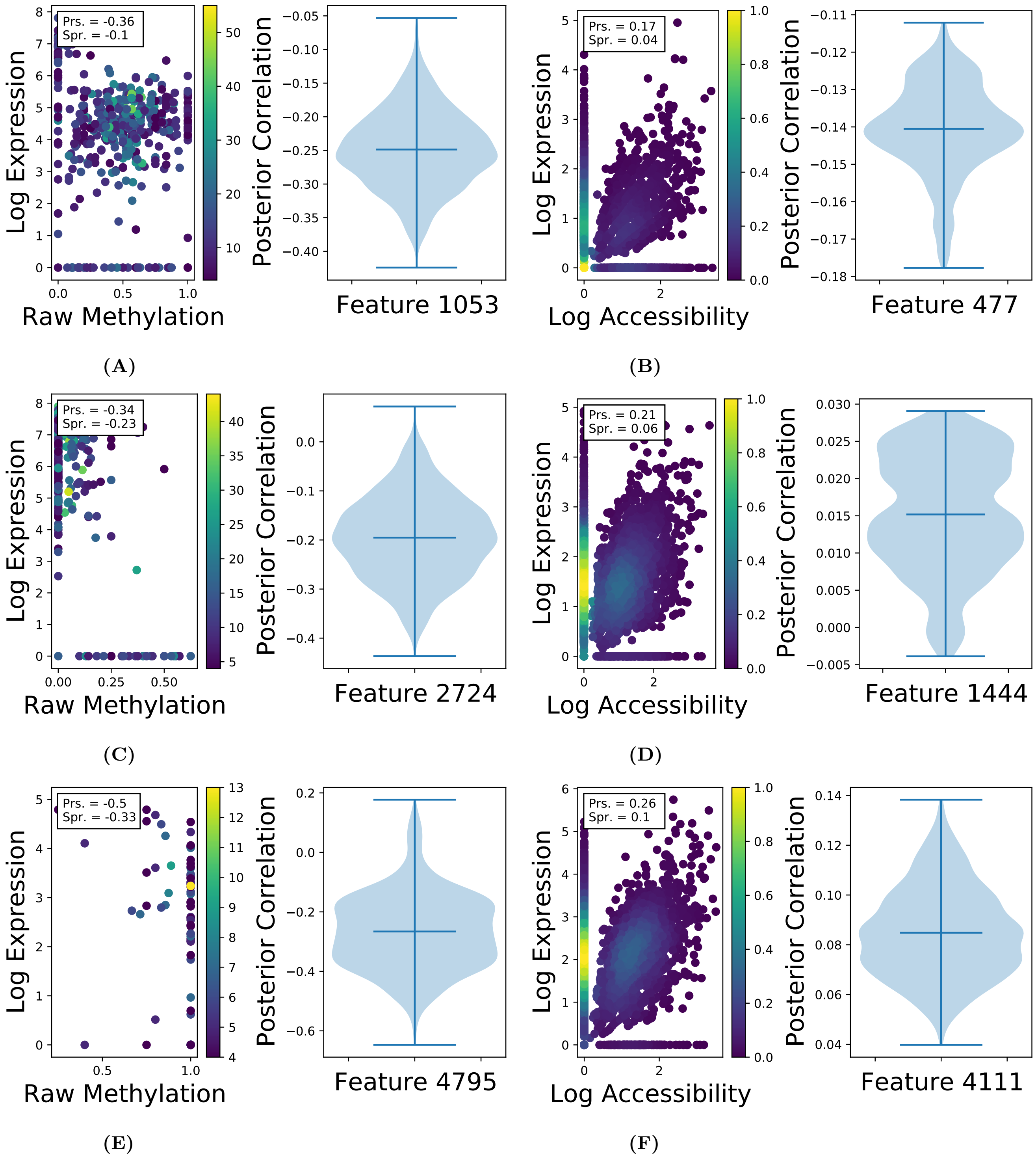

Fig B. Example where only Pearson identifies the feature's association as significant in mESC and mEBC datasets. In all figures the left part is a scatter plot of feature's raw data and the right part part is the posterior correlation as inferred by SCRaPL. In features from [1] data (ie. (BA), (BC) and (BE)) each dot of the scatter plot represents a cell reading, color-coded by CpG coverage and the expression axis is in $log(1 + x)$ scale. In features from mEBC data (ie. (BB), (BD) and (BF)) each dot of the scatter plot represents a cell reading, color-coded by space occupation and the accessibility/expression axis are in $log(1 + x)$ scale.

3 Classified as significant by EFDR and insignificant by FDR

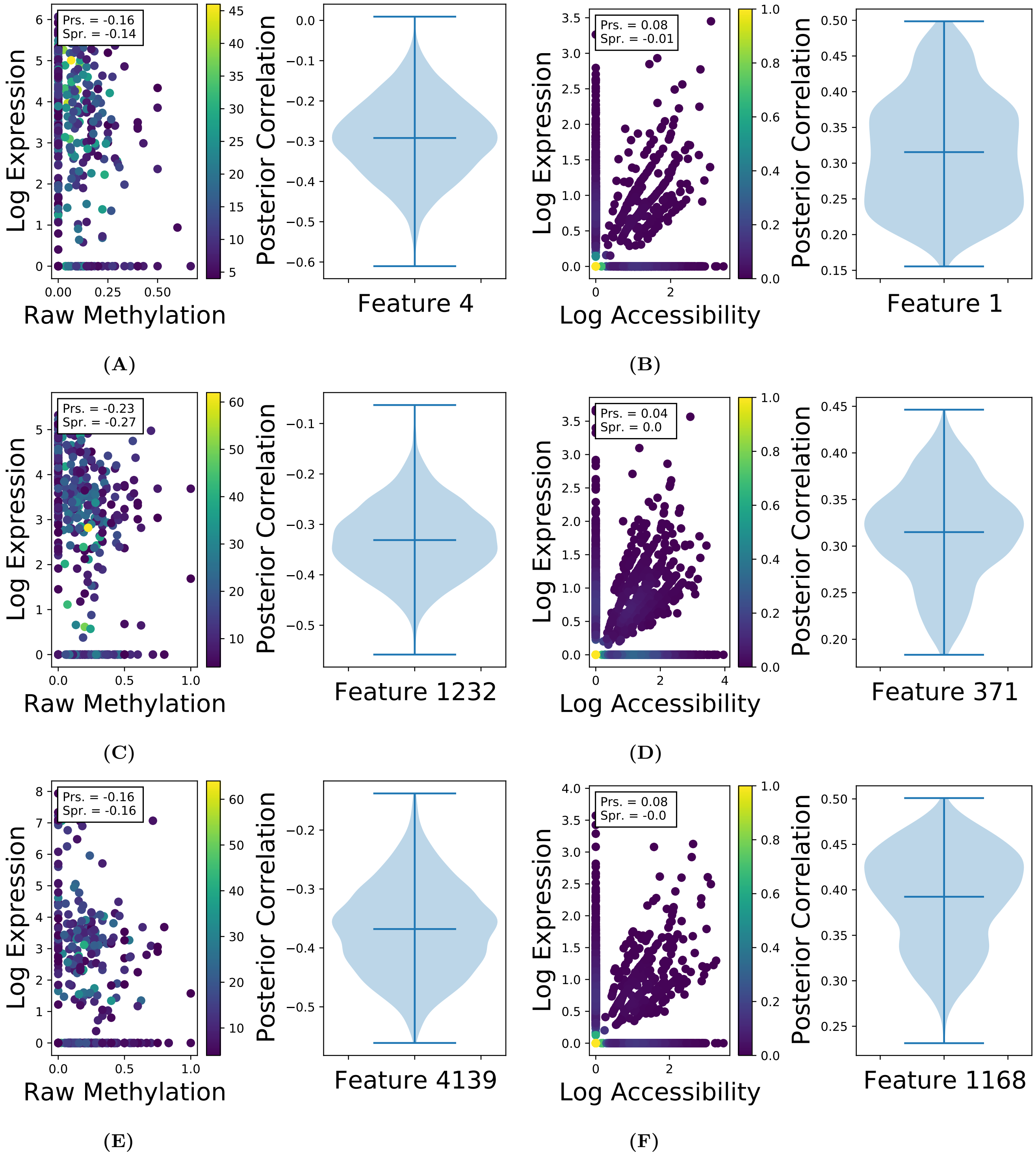

Fig C. Example where only SCRaPL identifies the feature's association as significant in mESC and mEBC datasets. In all figures the left part is a scatter plot of feature's raw data and the right part part is the posterior correlation as inferred by SCRaPL. In features from [1] data (ie. (CA), (CC) and (CE)) each dot of the scatter plot represents a cell reading, color-coded by CpG coverage and the expression axis is in $log(1+x)$ scale. In features from mEBC data (ie. (CB), (CD) and (CF)) each dot of the scatter plot represents a cell reading, color-coded by space occupation and the accessibility/expression axis are in $log(1+x)$ scale.

4 Posterior correlation inference for common genes in bibliography

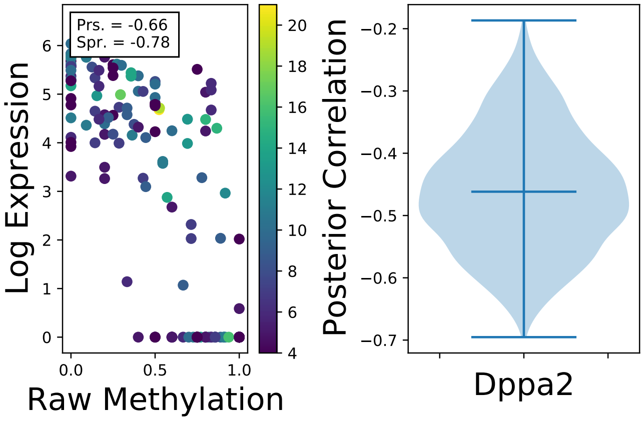

(A)

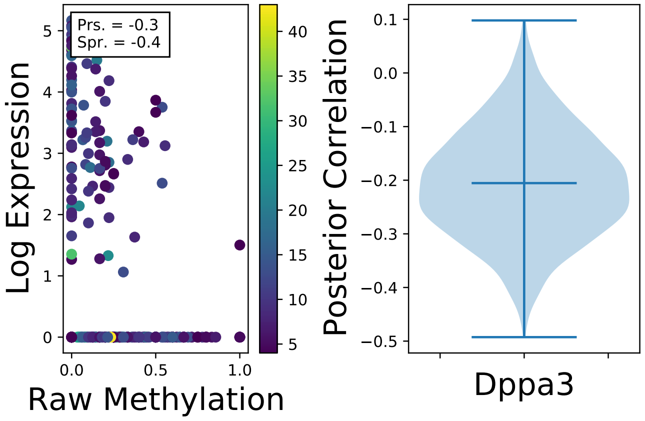

(B)

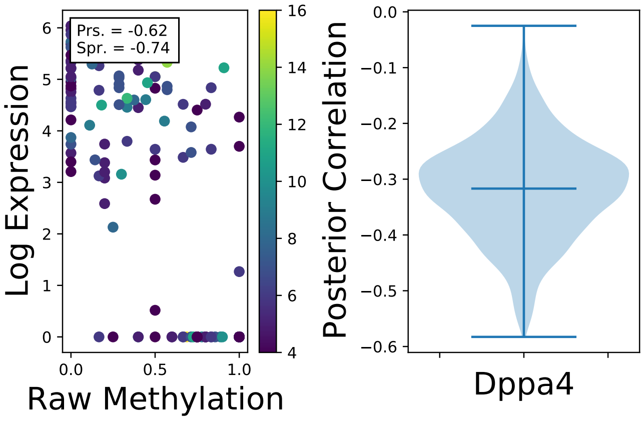

(C)

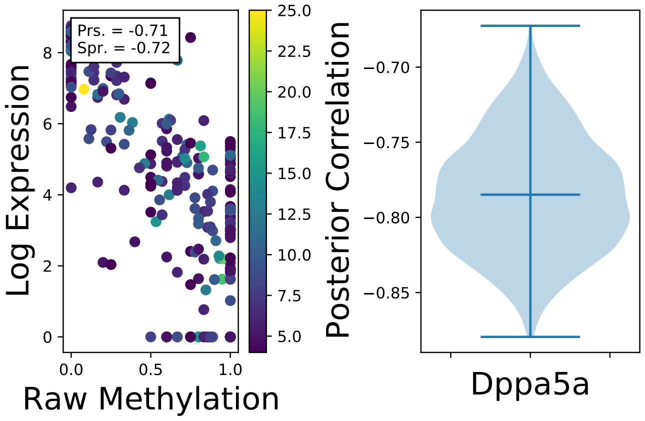

(D)

Fig D. Raw data and posterior gene correlation for selected members of the Dppa gene family, inferred with SCRaPL.

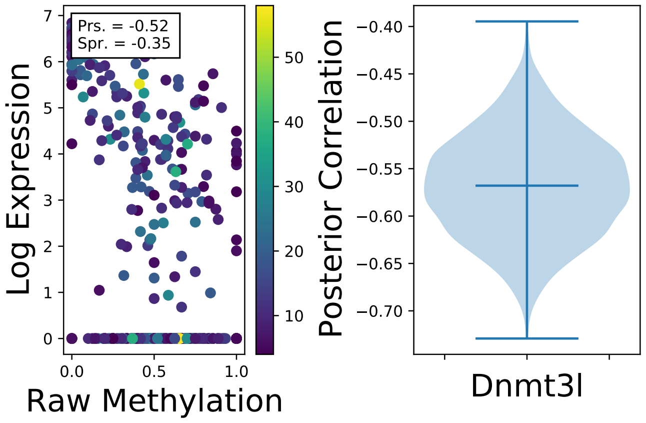

(A)

Fig E. Raw data and posterior gene correlation for selected members of the Dnmt gene family, inferred with SCRaPL.

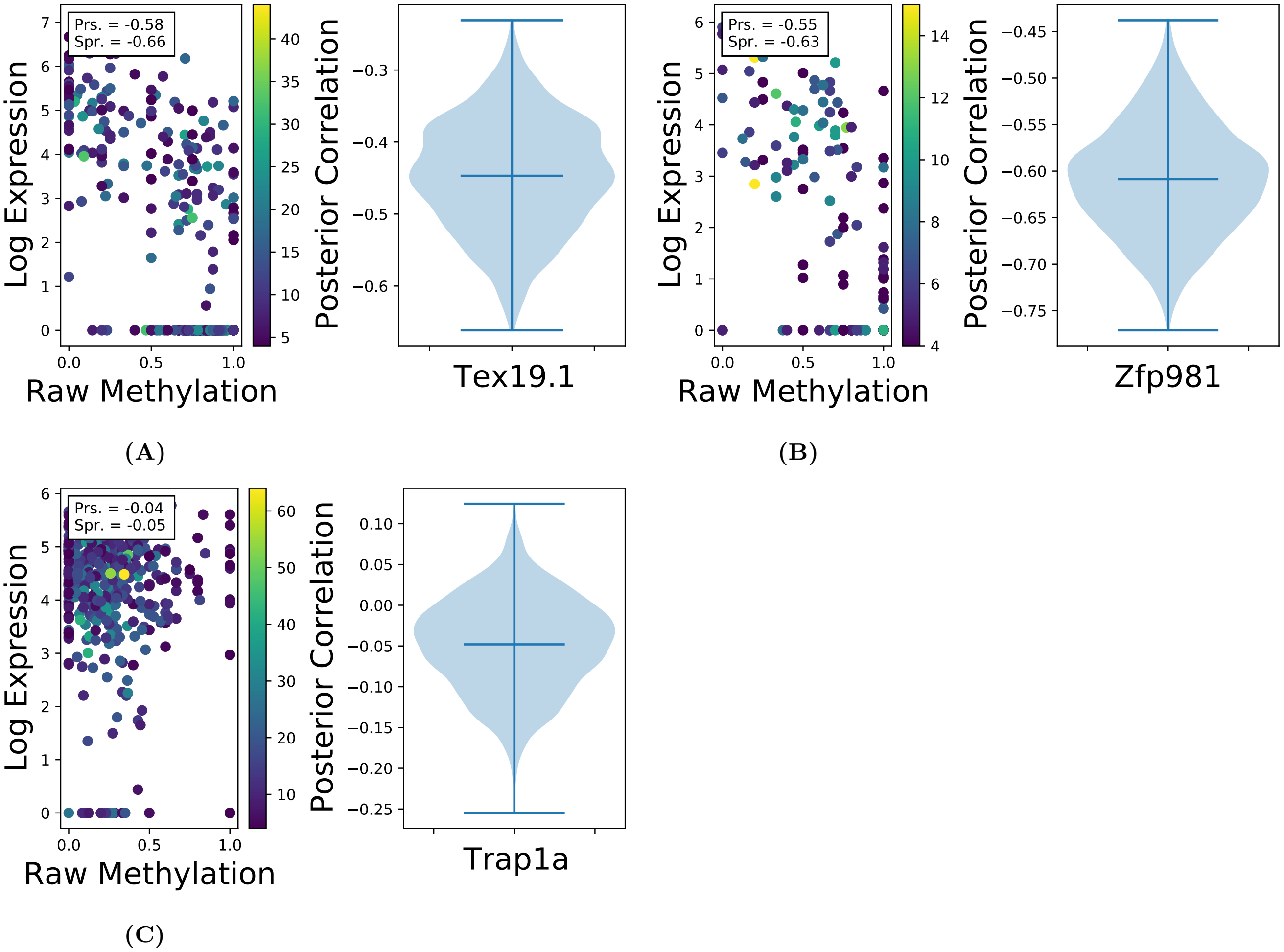

Fig F. Raw data and posterior gene correlation for other genes in [1] that have been partially or not studied, inferred with SCRaPL.

References

1. Argelaguet R, Clark SJ, Mohammed H, Stapel LC, Krueger C, Kapourani CA, et al. Multi-omics profiling of mouse gastrulation at single-cell resolution. Nature. 2019;576(7787):487-91.
