# Supplementary material for: SCRaPL: A Bayesian hierarchical framework for detecting technical associates in single cell multiomics data
Source: PLoS Comput Biol. 2022 Jun 21;18(6):e1010163. doi: 10.1371/journal.pcbi.1010163 (PMC9249169; doi:10.1371/journal.pcbi.1010163)
Supplement: S7 Text — The complete gene set enrichment analysis using DAVID is provided. (PDF) [file pcbi.1010163.s007.pdf]

S7 Text: Gene Set Enrichment Analysis.

SCRaPL: A Bayesian hierarchical framework for detecting technical associates in single cell multiomics data.

Christos Maniatis[1*], Catalina A. Vallejos[2,3*], Guido Sanguinetti[4,1*]

1 School of Informatics, The University of Edinburgh, Edinburgh, UK
2 The Alan Turing Institute, London,UK
3 MRC Human Genetics Unit, Institute of Genetics and Cancer, Western General Hospital, The University of Edinburgh, , Edinburgh, UK
4 International School for Advanced Studies (SISSA-ISA), Trieste, Italy

* s1315538@sms.ed.ac.uk(CM);* catalina.vallejos@ed.ac.uk(CAV);* gsanguin@sissa.it(GS)

To get a round understanding of the genes with strong regulatory action identified by alternative approaches, we carry our Gene Set Enrichment Analysis (GSEA) analysis using DAVID [1]. This process helps us to identify important biological processes by looking at over-represented genes in a starting pool of genetic markers. Using SCRaPL correlation outcomes we can determine a lists of biological outcomes that practitioners would discover. The γ threshold for SCRaPL was set to 0.205 or 90% quantile of the folded distribution constructed by correlation samples of the permuted dataset.

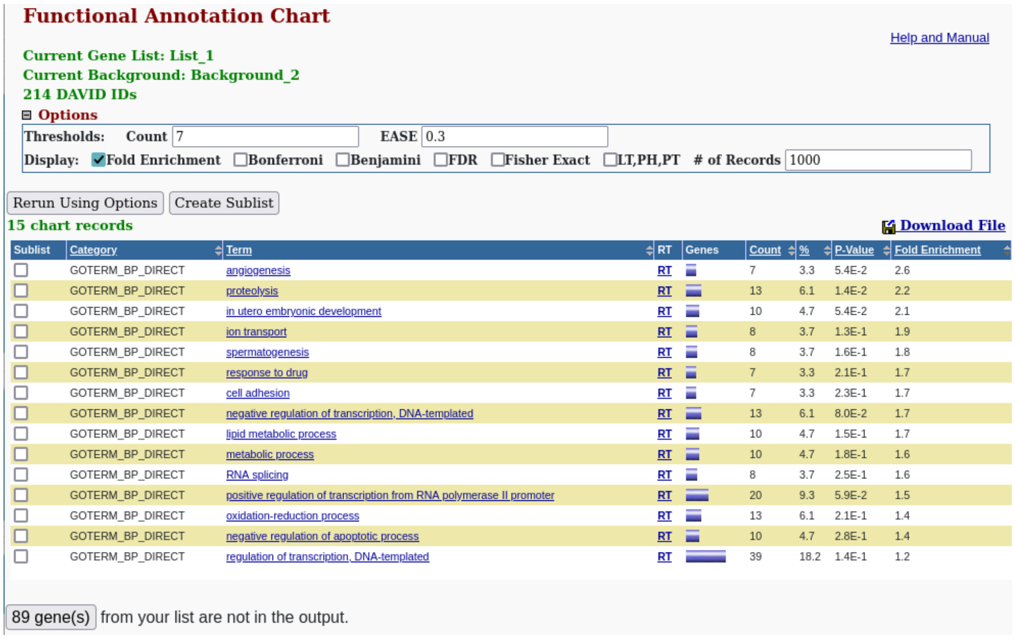

Functional Annotation Chart

Help and Manual

Current Gene List: List_1
Current Background: Background_2
214 DAVID IDs
Options
Thresholds: **Count** 7 **EASE** 0.3
Display: ☑ **Fold Enrichment** ☐ **Bonferroni** ☐ **Benjamini** ☐ **FDR** ☐ **Fisher Exact** ☐ **LT,PH,PT** **# of Records** 1000

Rerun Using Options | Create Sublist

15 chart records **Download File**

Sublist	Category	Term	RT	Genes	Count	%	P-Value	Fold Enrichment
☐	GOTERM_BP_DIRECT	angiogenesis	RT		7	3.3	5.4E-2	2.6
☐	GOTERM_BP_DIRECT	proteolysis	RT		13	6.1	1.4E-2	2.2
☐	GOTERM_BP_DIRECT	in utero embryonic development	RT		10	4.7	5.4E-2	2.1
☐	GOTERM_BP_DIRECT	ion transport	RT		8	3.7	1.3E-1	1.9
☐	GOTERM_BP_DIRECT	spermatogenesis	RT		8	3.7	1.6E-1	1.8
☐	GOTERM_BP_DIRECT	response to drug	RT		7	3.3	2.1E-1	1.7
☐	GOTERM_BP_DIRECT	cell adhesion	RT		7	3.3	2.3E-1	1.7
☐	GOTERM_BP_DIRECT	negative regulation of transcription, DNA-templated	RT		13	6.1	8.0E-2	1.7
☐	GOTERM_BP_DIRECT	lipid metabolic process	RT		10	4.7	1.5E-1	1.7
☐	GOTERM_BP_DIRECT	metabolic process	RT		10	4.7	1.8E-1	1.6
☐	GOTERM_BP_DIRECT	RNA splicing	RT		8	3.7	2.5E-1	1.6
☐	GOTERM_BP_DIRECT	positive regulation of transcription from RNA polymerase II promoter	RT		20	9.3	5.9E-2	1.5
☐	GOTERM_BP_DIRECT	oxidation-reduction process	RT		13	6.1	2.1E-1	1.4
☐	GOTERM_BP_DIRECT	negative regulation of apoptotic process	RT		10	4.7	2.8E-1	1.4
☐	GOTERM_BP_DIRECT	regulation of transcription, DNA-templated	RT		39	18.2	1.4E-1	1.2

89 gene(s) from your list are not in the output.

(A)

Fig A. GO analysis with features detected by SCRaPL.

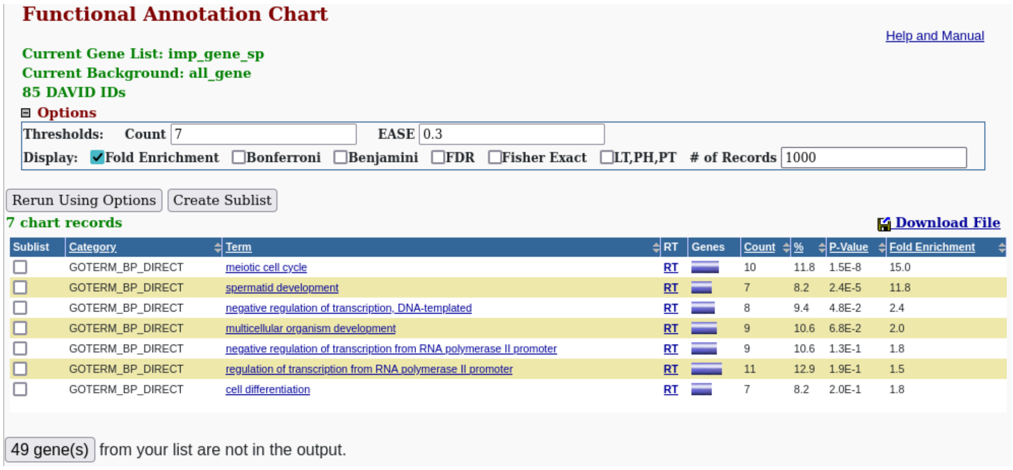

Sublist	Category	Term	RT	Genes	Count	%	P-Value	Fold Enrichment
	GOTERM_BP_DIRECT	meiotic cell cycle	RT		10	11.8	1.5E-8	15.0
	GOTERM_BP_DIRECT	spermatid development	RT		7	8.2	2.4E-5	11.8
	GOTERM_BP_DIRECT	negative regulation of transcription, DNA-templated	RT		8	9.4	4.8E-2	2.4
	GOTERM_BP_DIRECT	multicellular organism development	RT		9	10.6	6.8E-2	2.0
	GOTERM_BP_DIRECT	negative regulation of transcription from RNA polymerase II promoter	RT		9	10.6	1.3E-1	1.8
	GOTERM_BP_DIRECT	regulation of transcription from RNA polymerase II promoter	RT		11	12.9	1.9E-1	1.5
	GOTERM_BP_DIRECT	cell differentiation	RT		7	8.2	2.0E-1	1.8

(A)

Fig B. GO analysis with features detected by Spearman correlation.

The detected biological presented in Figs A,B have at 7 or more genes associated with them and maximum p-value 0.3. Then processes were sorted based on their enrichment score (ie. how many times larger is the detected set compared to a random subset of the pool of genes linked to a particular biological process). SCRaPL has detected many processes directly linked to regulation of transcription and regulation of transcription in promoter regions (something expected as we are looking at promoters of a methylation-expression pair at early development where methylation plays a crucial role). Apart from them there are also biological processes linked to development, like in utero embryonic development and angiogenesis with high enrichment score. Spearman also detects processes linked to transcription regulation like negative regulation of transcription or meiotic cell cycle as seen in Fig B. However no process with immediate links to development appears. For the exact same filtering parameter and genes detected with Pearson correlation, the enrichment would link genes to ”regulation of transcription” with enrichment score 1.5.

References

1. Sherman BT, Lempicki RA, et al. Systematic and integrative analysis of large gene lists using DAVID bioinformatics resources. Nature protocols. 2009;4(1):44.
