# Supplementary material for: SCRaPL: A Bayesian hierarchical framework for detecting technical associates in single cell multiomics data
Source: PLoS Comput Biol. 2022 Jun 21;18(6):e1010163. doi: 10.1371/journal.pcbi.1010163 (PMC9249169; doi:10.1371/journal.pcbi.1010163)
Supplement: S8 Text — We demonstrate that SCRaPL’s expression likelihood serves as valid alternative as it exhibits the over-dispersion property that practitioners seek. (PDF) [file pcbi.1010163.s008.pdf]

S8 Text: Connecting SCRaPL error model to likelihoods currently employed by practitioners.

SCRaPL: A Bayesian hierarchical framework for detecting technical associates in single cell multiomics data.

Christos Maniatis[1*], Catalina A. Vallejos[2,3*], Guido Sanguinetti[4,1*]

1 School of Informatics, The University of Edinburgh, Edinburgh, UK
2 The Alan Turing Institute, London,UK
3 MRC Human Genetics Unit, Institute of Genetics and Cancer, Western General Hospital, The University of Edinburgh, , Edinburgh, UK
4 International School for Advanced Studies (SISSA-ISA), Trieste, Italy

* s1315538@sms.ed.ac.uk(CM);* catalina.vallejos@ed.ac.uk(CAV);* gsanguin@sissa.it(GS)

In the field of transcriptomics it is widely accepted that over-dispersion is an important feature of state of the art models [1]. Following the example of several papers (e.g, [1; 2; 3]) where they provide evidence that their model can handle excessive zeros by using over-dispersion, we demonstrate that zero-inflated Poisson is also a valid alternative as the over-dispersion property exists. For the sake of completeness we provide mean and variance formulas for the binomial posterior.

1 Zero-inflated Poisson

$$\mathbb{P}\left(y\right) = \begin{cases} \int_{-\infty}^{\infty} \pi + (1-\pi)e^{-ae^x}\mathbb{N}\left(x,\mu,\sigma^2\right)dx, \textbf{if } y=0 \\ \int_{-\infty}^{\infty} \frac{1-\pi}{y!}a^y e^{yx-ae^x}\mathbb{N}\left(x,\mu,\sigma^2\right)dx, \textbf{else} \end{cases} \tag{1}$$

$$\begin{aligned}\mathbb{E}\left(y\right) &= \sum_{y=0}^{\infty} y\mathbb{P}\left(y\right) = \sum_{y=1}^{\infty} y\mathbb{P}\left(y\right) = \sum_{y=1}^{\infty} y\frac{1-\pi}{y!}a^y \int_{-\infty}^{\infty} e^{yx-ae^x}\mathbb{N}\left(x,\mu,\sigma^2\right)dx \\ &= \sum_{y=1}^{\infty} \frac{1-\pi}{(y-1)!}a^y \int_{-\infty}^{\infty} e^{yx-ae^x}\mathbb{N}\left(x,\mu,\sigma^2\right)dx \\ &= a(1-\pi)\int_{-\infty}^{\infty} e^x e^{-ae^x}\mathbb{N}\left(x,\mu,\sigma^2\right)\sum_{y=1}^{\infty} a^{y-1}\frac{e^{(y-1)x}}{(y-1)!}dx \\ &= a(1-\pi)\int_{-\infty}^{\infty} e^x e^{-ae^x}\mathbb{N}\left(x,\mu,\sigma^2\right)\sum_{y=0}^{\infty} a^{y}\frac{e^{yx}}{y!}dx \\ &= a(1-\pi)\int_{-\infty}^{\infty} e^x e^{-ae^x}\mathbb{N}\left(x,\mu,\sigma^2\right)e^{ae^x}dx \\ &= a(1-\pi)\int_{-\infty}^{\infty} e^x \mathbb{N}\left(x,\mu,\sigma^2\right)dx = a(1-\pi)e^{\mu+\frac{\sigma^2}{2}}\end{aligned}$$

Hence,

$$\mathbb{E}\left(y\right) = a(1-\pi)e^{\mu+\frac{\sigma^2}{2}} = m \tag{2}$$

$$\mathbb{E}\left(y^2\right)=\sum_{y=0}^{\infty}y^2\mathbb{P}\left(y\right)=\sum_{y=1}^{\infty}y^2\mathbb{P}\left(y\right)$$

$$=\sum_{y=1}^{\infty}y^2\frac{1-\pi}{y!}\int_{-\infty}^{\infty}a^y e^{yx-ae^x}\mathbb{N}\left(x,\mu,\sigma^2\right)dx=\sum_{y=1}^{\infty}y\frac{1-\pi}{(y-1)!}\int_{-\infty}^{\infty}a^y e^{yx-ae^x}\mathbb{N}\left(x,\mu,\sigma^2\right)dx$$

$$=a(1-\pi)\int_{-\infty}^{\infty}e^x e^{-ae^x}\mathbb{N}\left(x,\mu,\sigma^2\right)\sum_{y=0}^{\infty}\frac{(y+1)a^y e^{yx}}{y!}dx$$

$$=a(1-\pi)\int_{-\infty}^{\infty}e^x e^{-ae^x}\mathbb{N}\left(x,\mu,\sigma^2\right)\sum_{y=0}^{\infty}\frac{ya^y e^{yx}}{y!}dx+a(1-\pi)\int_{-\infty}^{\infty}e^x e^{-ae^x}\mathbb{N}\left(x,\mu,\sigma^2\right)\sum_{y=0}^{\infty}\frac{a^y e^{yx}}{y!}dx$$

$$=a(1-\pi)\int_{-\infty}^{\infty}e^x e^{-ae^x}\mathbb{N}\left(x,\mu,\sigma^2\right)\sum_{y=1}^{\infty}\frac{a^y e^{yx}}{(y-1)!}dx+a(1-\pi)\int_{-\infty}^{\infty}e^x\mathbb{N}\left(x,\mu,\sigma^2\right)dx$$

$$=a^2(1-\pi)\int_{-\infty}^{\infty}e^{2x}e^{-ae^x}\mathbb{N}\left(x,\mu,\sigma^2\right)\sum_{y=0}^{\infty}\frac{a^y e^{yx}}{y!}dx+a(1-\pi)\int_{-\infty}^{\infty}e^x\mathbb{N}\left(x,\mu,\sigma^2\right)dx$$

$$=a^2(1-\pi)\int_{-\infty}^{\infty}e^{2x}\mathbb{N}\left(x,\mu,\sigma^2\right)dx+a(1-\pi)\int_{-\infty}^{\infty}e^x\mathbb{N}\left(x,\mu,\sigma^2\right)dx=a(1-\pi)\left[ae^{2\mu+2\sigma^2}+e^{\mu+\frac{\sigma^2}{2}}\right]$$

$$\mathbb{V}\left(y\right)=\mathbb{E}\left(y^2\right)-\mathbb{E}\left(y\right)^2=a(1-\pi)e^{\mu+\frac{\sigma^2}{2}}\left(1+ae^{\mu+\frac{3\sigma^2}{2}}\right)-a^2(1-\pi)^2e^{2\mu+\sigma^2}$$

$$=a(1-\pi)e^{\mu+\frac{\sigma^2}{2}}\left[1+ae^{\mu+\frac{3\sigma^2}{2}}-a(1-\pi)e^{\mu+\frac{\sigma^2}{2}}\right]$$

$$=a(1-\pi)e^{\mu+\frac{\sigma^2}{2}}\left[1+ae^{\mu+\frac{\sigma^2}{2}}\left(e^{\sigma^2}-1+\pi\right)\right]=m\left(1+m\frac{e^{\sigma^2}-1+\pi}{1-\pi}\right)$$

Hence,

$$\mathbb{V}\left(y\right)=m\left(1+m\frac{e^{\sigma^2}-1+\pi}{1-\pi}\right)\tag{3}$$

2 Binomial

$$\mathbb{P}(k)=\int_{-\infty}^{\infty}\binom{n}{k}\Phi(x)^k\left(1-\Phi(x)\right)^{n-k}\mathbb{N}\left(x,\mu,\sigma^2\right)dx\tag{4}$$

$$\mathbb{E}\left(k\right)=\sum_{k=0}^{n}k\mathbb{P}(k)=\int_{-\infty}^{\infty}\mathbb{N}\left(x,\mu,\sigma^2\right)\sum_{k=0}^{n}k\binom{n}{k}\Phi(x)^k\left(1-\Phi(x)\right)^{n-k}dx$$

$$=n\int_{-\infty}^{\infty}\Phi(x)\mathbb{N}\left(x,\mu,\sigma^2\right)dx=n\Phi\left(\frac{\mu}{\sqrt{1+\sigma^2}}\right)$$

Hence,

$$\mathbb{E}\left(k\right)=n\Phi\left(\frac{\mu}{\sqrt{1+\sigma^2}}\right)\tag{5}$$

$$\mathbb{E}\left(k(k-1)\right)=\sum_{k=0}^{n}k(k-1)\mathbb{P}(k)=\int_{-\infty}^{\infty}\mathbb{N}\left(x,\mu,\sigma^2\right)\sum_{k=0}^{n}k(k-1)\binom{n}{k}\Phi(x)^k\left(1-\Phi(x)\right)^{n-k}dx$$

$$=n(n-1)\int_{-\infty}^{\infty}\Phi(x)^2\mathbb{N}\left(x,\mu,\sigma^2\right)=n(n-1)\left[\Phi\left(\frac{\mu}{\sqrt{1+\sigma^2}}\right)-2T\left(\frac{\mu}{\sqrt{1+\sigma^2}},\frac{1}{\sqrt{1+2\sigma^2}}\right)\right],$$

$$T\left(h,a\right)=\mathbb{N}(h,0,1)\int_{0}^{a}\frac{\mathbb{N}(hx,0,1)}{1+x^2}dx$$

$$\mathbb{V}\left(k\right)=\mathbb{E}\left(k^2\right)-\mathbb{E}\left(k\right)^2=\mathbb{E}\left(k(k-1)\right)+\mathbb{E}\left(k\right)-\mathbb{E}\left(k\right)^2$$

$$=n(n-1)\left[\Phi\left(\frac{\mu}{\sqrt{1+\sigma^2}}\right)-2T\left(\frac{\mu}{\sqrt{1+\sigma^2}},\frac{1}{\sqrt{1+2\sigma^2}}\right)\right]+n\Phi\left(\frac{\mu}{\sqrt{1+\sigma^2}}\right)-n^2\Phi\left(\frac{\mu}{\sqrt{1+\sigma^2}}\right)^2$$

$$=n^2\Phi\left(\frac{\mu}{\sqrt{1+\sigma^2}}\right)\left[1-\Phi\left(\frac{\mu}{\sqrt{1+\sigma^2}}\right)\right]-2n(n-1)T\left(\frac{\mu}{\sqrt{1+\sigma^2}},\frac{1}{\sqrt{1+2\sigma^2}}\right)$$

hence,

$$\mathbb{V}\left(k\right)=n^2\Phi\left(\frac{\mu}{\sqrt{1+\sigma^2}}\right)\left[1-\Phi\left(\frac{\mu}{\sqrt{1+\sigma^2}}\right)\right]-2n(n-1)T\left(\frac{\mu}{\sqrt{1+\sigma^2}},\frac{1}{\sqrt{1+2\sigma^2}}\right)$$

References

1. Svensson V. Droplet scRNA-seq is not zero-inflated. Nature Biotechnology. 2020;38(2):147-50.
2. Vallejos CA, Richardson S, Marioni JC. Beyond comparisons of means: understanding changes in gene expression at the single-cell level. Genome biology. 2016;17(1):1-14.
3. He L, Kulminski A. NEBULA: a fast negative binomial mixed model for differential expression and co-expression analyses of large-scale multi subject single-cell data. bioRxiv. 2020.
