# Supplementary material for: SCRaPL: A Bayesian hierarchical framework for detecting technical associates in single cell multiomics data
Source: PLoS Comput Biol. 2022 Jun 21;18(6):e1010163. doi: 10.1371/journal.pcbi.1010163 (PMC9249169; doi:10.1371/journal.pcbi.1010163)
Supplement: S9 Text — We give a thorough description of the hypothesis testing done to identify regions with strong regulatory action. (PDF) [file pcbi.1010163.s009.pdf]

S9 Text: Null Hypothesis Testing.

SCRaPL: A Bayesian hierarchical framework for detecting technical associates in single cell multiomics data.

Christos Maniatis[1*], Catalina A. Vallejos[2,3*], Guido Sanguinetti[4,1*]

1 School of Informatics, The University of Edinburgh, Edinburgh, UK
2 The Alan Turing Institute, London,UK
3 MRC Human Genetics Unit, Institute of Genetics and Cancer, Western General Hospital, The University of Edinburgh, , Edinburgh, UK
4 International School for Advanced Studies (SISSA-ISA), Trieste, Italy

* s1315538@sms.ed.ac.uk(CM);* catalina.vallejos@ed.ac.uk(CAV);* gsanguin@sissa.it(GS)

1 Pearson

In most settings where Pearson correlation with hypothesis testing is applied, the aim is to determine whether the estimated value of correlation is generated from an uncorrelated bivariate normal distribution. Here we are interested to investigate the more complicated null hypothesis that the data generating correlation lives in an interval around 0. Hence the first step is to determine the distribution of sampled Pearson correlations r given true correlation ρ in a correlated bivariate normal distribution. According to [1] that distribution is:

$$f(r,\rho) = \frac{(n-2)\Gamma(n-1)(1-\rho^2)^{\frac{n-1}{2}}(1-r^2)^{\frac{n-2}{4}}}{\sqrt{2\pi}\Gamma(n-\frac{1}{2})(1-\rho r)^{n-\frac{3}{2}}}\,{}_2F_1\left(\frac{1}{2},\frac{1}{2},\frac{2n-1}{2},\frac{1+r\rho}{2},\right) \tag{1}$$

where Γ is the gamma function and ${}_2F_1$ is the Gaussian hypergeometric function. In the special case of $\rho = 0$ we get student a reparametrized version of student t-distribution. However we are interested in the null distribution of correlations with magnitude below a threshold γ_{prs}. The get it, we integrate $f(r,\rho)$ over that range $[-\gamma_{prs}, \gamma_{prs}]$.

$$p(r) = \frac{1}{\mathbb{Z}}\int_{-\gamma_{prs}}^{\gamma_{prs}} f(r,\rho)d\rho \tag{2}$$

Where $\mathbb{Z} = \int_{-1}^{1}\int_{-\gamma_{prs}}^{\gamma_{prs}} f(r,\rho)d\rho dr$. Integrating $f(r,\rho)$ over ρ is not trivial as there is not closed form solution. Hence we resort to numerical integration using Matlab's/Python's built in function. The p-value under the null hypothesis for a Pearson correlation estimate p_{cor} is

$$\mathbb{P}(R \geq |p_{cor}|) = \int_{-1}^{-|p_{cor}|} p(r)dr + \int_{|p_{cor}|}^{1} p(r)dr = 2\int_{-1}^{-|p_{cor}|} p(r)dr \tag{3}$$

To simplify this integral and get the simplified expression of the right hand side, we use that $f(r,\rho) = f(-r,-\rho)$. Type I error is controlled with standard FDR [2] as in the case of $\gamma_{prs} = 0$.

2 SCRaPL

The aim of SCRaPL is to identify genomic regions with strong correlation across molecular layers using feature specific posterior correlation. Mathematically this is done by estimating the probability of correlation's magnitude (ie. $|\rho_j|$) being above a threshold γ.

$$p_j(\gamma) = \mathbb{P}(|\rho_j| \geq \gamma) \tag{4}$$

If $p_j(\gamma)$ is larger than a threshold a then correlation on genomic region j is labeled statistically significant. To estimate γ we have a data driven approach in place. More precisely, we look various quantiles in negative control data. Using γ we calibrate α such that EFDR is below 10%. Since $p_j(\gamma)$ is a cumulative density function, under the null hypothesis it is uniformly distributed. Hence we apply the same procedure in the original and negative control data and compare detection rates. This test becomes problematic in case $\gamma = 0$ as $p_j(\gamma) = 1$ for every j. In this case we apply the rule from [3] based on the maximum posterior probabilities associated to the one-sided hypothesis $\rho_j > 0$ and $\rho_j < 0$, mathematically summarized as follows:

$$2\max(\pi_j, 1 - \pi_j) - 1 > a, \ \textbf{with} \ \pi_j = \mathbb{P}(\rho_j \geq 0) \tag{5}$$

Parameter a is calibrated such that EFDR is below 10%. The max-rule in equation 5 is uniformly distributed under the null hypothesis. This limitation of this rule is that results are correct for the case of symmetric around 0 posterior correlation distributions. Therefore, we use it here as an approximation.

References

1. Hotelling H. New light on the correlation coefficient and its transforms. Journal of the Royal Statistical Society Series B (Methodological). 1953;15(2):193-232.

2. Benjamini Y, Hochberg Y. Controlling the false discovery rate: a practical and powerful approach to multiple testing. Journal of the Royal statistical society: series B (Methodological). 1995;57(1):289-300.

3. Bochkina N, Richardson S. Tail posterior probability for inference in pairwise and multiclass gene expression data. Biometrics. 2007;63(4):1117-25.
