# Supplementary material for: SCRaPL: A Bayesian hierarchical framework for detecting technical associates in single cell multiomics data
Source: PLoS Comput Biol. 2022 Jun 21;18(6):e1010163. doi: 10.1371/journal.pcbi.1010163 (PMC9249169; doi:10.1371/journal.pcbi.1010163)
Supplement: S10 Text — We provide evidence of SCRaPL’s scaling as a function of problem size. (PDF) [file pcbi.1010163.s010.pdf]

S10 Text: Efficiency Analysis.

SCRaPL: A Bayesian hierarchical framework for detecting technical associates in single cell multiomics data.

Christos Maniatis[1*], Catalina A. Vallejos[2,3*], Guido Sanguinetti[4,1*]

1 School of Informatics, The University of Edinburgh, Edinburgh, UK
2 The Alan Turing Institute, London,UK
3 MRC Human Genetics Unit, Institute of Genetics and Cancer, Western General Hospital, The University of Edinburgh, , Edinburgh, UK
4 International School for Advanced Studies (SISSA-ISA), Trieste, Italy

* s1315538@sms.ed.ac.uk(CM);* catalina.vallejos@ed.ac.uk(CAV);* gsanguin@sissa.it(GS)

As part of the newer Tensorflow implementation we performed efficiency analysis to understand model's scalability. In preliminary implementations we experimented with both Markov Chain Monte Carlo (MCMC) and Variational Inference (VI). Unfortunately VI did not yield accurate results so we abandoned it in favor of MCMC. Hence execution time as a function of scaling is summarized in the figure below.

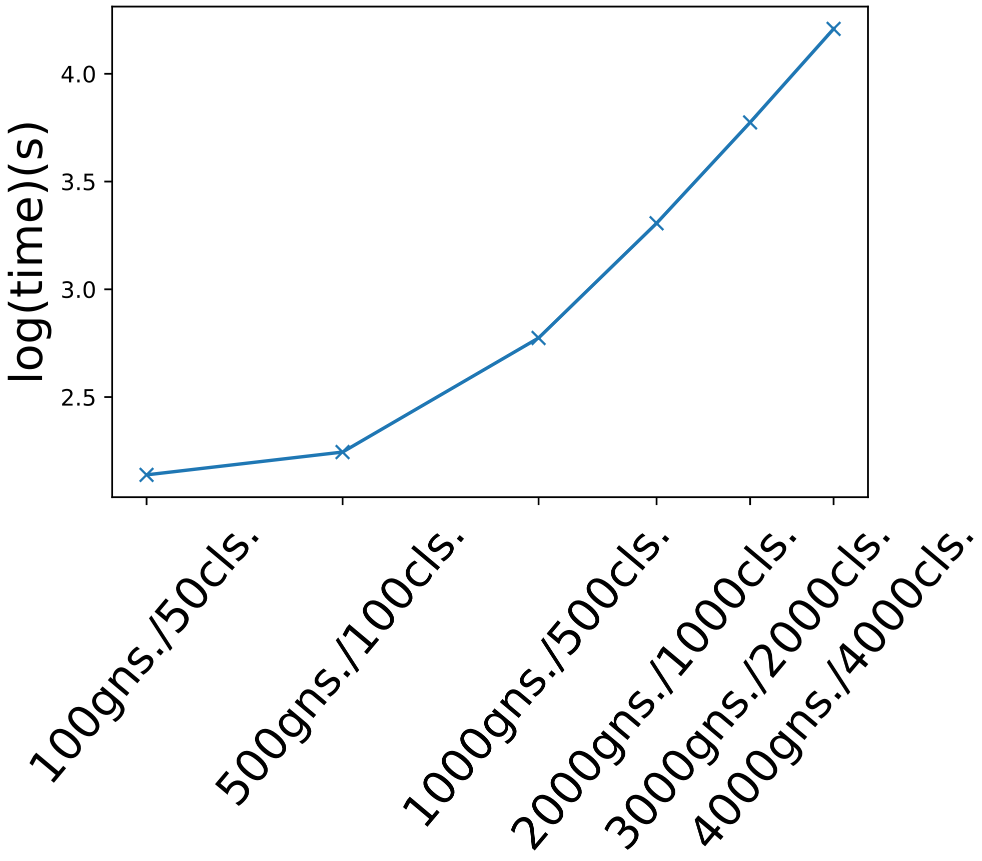

Fig A. Execution times as a function (features/cell numbers).
