# Supplementary material for: SCRaPL: A Bayesian hierarchical framework for detecting technical associates in single cell multiomics data
Source: PLoS Comput Biol. 2022 Jun 21;18(6):e1010163. doi: 10.1371/journal.pcbi.1010163 (PMC9249169; doi:10.1371/journal.pcbi.1010163)
Supplement: S11 Text — We provide additional figures to Fig 3D that demonstrate the different behavior that SCRaPL presents from Pearson/Spearman correlation when high zero inflation and low CpG coverage is present in data. (PDF) [file pcbi.1010163.s011.pdf]

S11 Text: Macroscopic analysis of SCRaPL's behavior compared to Pearson and Spearman Correlation.

SCRaPL: A Bayesian hierarchical framework for detecting technical associates in single cell multiomics data.

Christos Maniatis[1*], Catalina A. Vallejos[2,3*], Guido Sanguinetti[4,1*]

1 School of Informatics, The University of Edinburgh, Edinburgh, UK
2 The Alan Turing Institute, London,UK
3 MRC Human Genetics Unit, Institute of Genetics and Cancer, Western General Hospital, The University of Edinburgh, , Edinburgh, UK
4 International School for Advanced Studies (SISSA-ISA), Trieste, Italy

* s1315538@sms.ed.ac.uk(CM);* catalina.vallejos@ed.ac.uk(CAV);* gsanguin@sissa.it(GS)

In this section we provide evidence in favor of the arguments presented in section "SCRaPL associations are influenced by data sparsity and are 152 robust to outliers. ", about the differences between SCRaPL and different frequentist approaches considered in this work. More precisely, we argued that high CpG coverage and low inflation contribute in the reduction of discrepancy between SCRaPL's posterior correlation and Pearson/Spearman correlations. By regressing correlation discrepancy against these parameters we generate Fig A along with figure $3d$ presented in the main text which perfectly demonstrate the point.

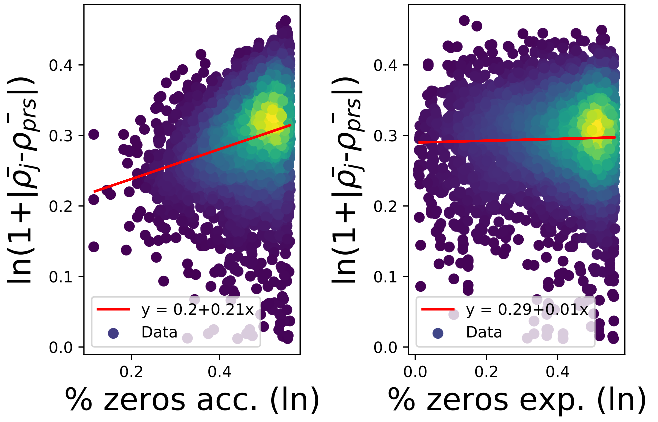

(A)

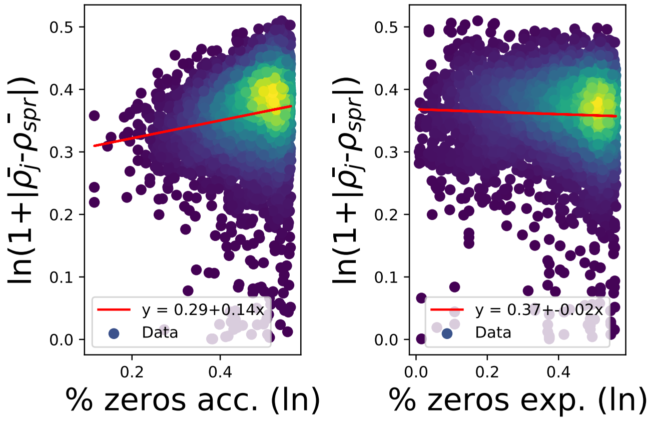

(B)

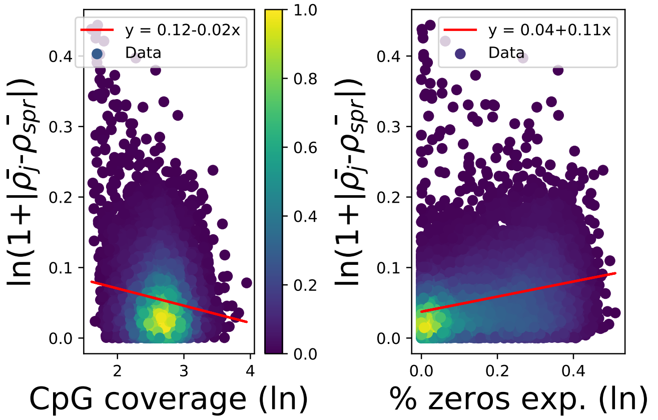

(C)

Fig A. SCRaPL's behavior compared to Pearson/Spearman correlation in micro and macro scale. (AA) Scatter plots to demonstrate the positive relationship between alternative correlation estimates (Pearson and SCRaPL) and % zeros in accessibility and expression respectively. (AB) Scatter plots to demonstrate the positive relationship between alternative correlation estimates (Spearman and SCRaPL) and % zeros in accessibility and expression respectively. (AC) Scatter plots to demonstrate the negative/positive relationship between alternative correlation estimates and CpG coverage/% zeros in expression respectively.
