# Supplementary material for: SCRaPL: A Bayesian hierarchical framework for detecting technical associates in single cell multiomics data
Source: PLoS Comput Biol. 2022 Jun 21;18(6):e1010163. doi: 10.1371/journal.pcbi.1010163 (PMC9249169; doi:10.1371/journal.pcbi.1010163)
Supplement: S12 Text — Figures summarizing scRNA and scATAC integration using posterior latent space samples. (PDF) [file pcbi.1010163.s012.pdf]

S12 Text: Stability of Denoising.

SCRaPL: A Bayesian hierarchical framework for detecting technical associates in single cell multiomics data.

Christos Maniatis[1*], Catalina A. Vallejos[2,3*], Guido Sanguinetti[4,1*]

1 School of Informatics, The University of Edinburgh, Edinburgh, UK
2 The Alan Turing Institute, London,UK
3 MRC Human Genetics Unit, Institute of Genetics and Cancer, Western General Hospital, The University of Edinburgh, , Edinburgh, UK
4 International School for Advanced Studies (SISSA-ISA), Trieste, Italy

* s1315538@sms.ed.ac.uk(CM);* catalina.vallejos@ed.ac.uk(CAV);* gsanguin@sissa.it(GS)

In the main text we looked the extend to which SCRaPL could be used as a denoising tool, to perform data integration at the level of latent space instead of raw data done by Seurat. Since denoising involves sampling from the posterior, we verify the consistency across different trials. As evident from Figs AA,AC and AE a tiny fraction of cells vary across trials, which implies that results are stable.

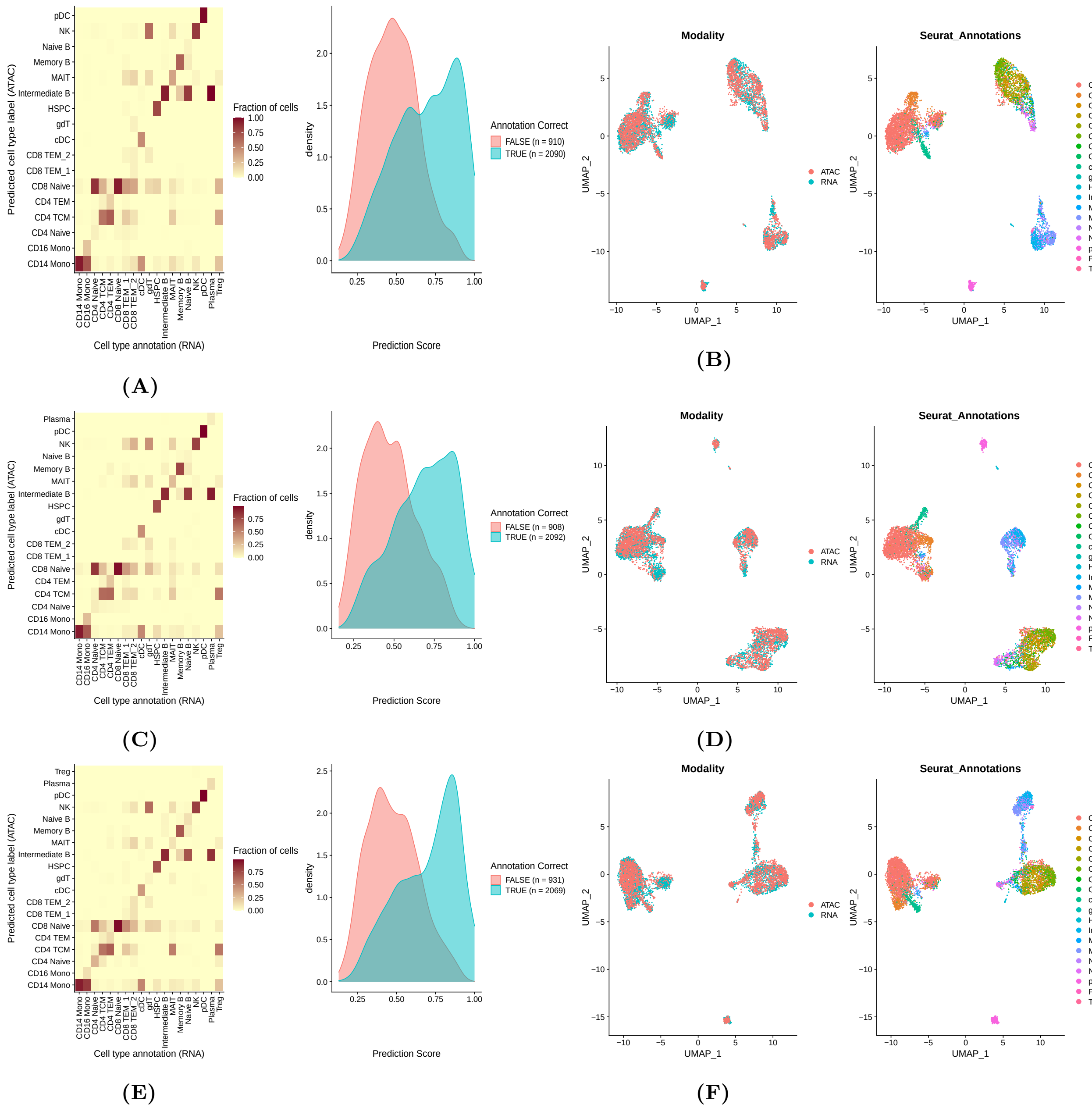

Fig A. Stability analysis of cell integration using SCRaPL as a denoising tool Panel summarizing label transfer from expression to accessibility in trials 2(AA), 3 (AC) and 4 (AE) along with respective latent plot visualizations (AB,AD,AE) of sc-RNA and scATAC
