# Supplementary material for: SCRaPL: A Bayesian hierarchical framework for detecting technical associates in single cell multiomics data
Source: PLoS Comput Biol. 2022 Jun 21;18(6):e1010163. doi: 10.1371/journal.pcbi.1010163 (PMC9249169; doi:10.1371/journal.pcbi.1010163)
Supplement: Attachment — Submitted filename: 117169_1_rebuttal_2136573_qwwftc.pdf [file pcbi.1010163.s013.pdf]

Description of the planned revisions

Reviewer 1

This paper proposes a noise-aware approach SCRaPL for modelling the associations of single cell multi-omic data. For gene expression, it uses Poisson-lognormal model. For DNAm data, it uses Binomial noise model which explicitly takes into account the average within the region. The Bayesian hierarchical framework employed by SCRaPL could achieve higher sensitivity and better robustness in identifying correlations, and also offer a template for the application of more complex analysis techniques to multi-omics data. The symbols of this paper are a little bit confusing, and I suggest authors to carefully check them.

We thank the reviewer for his/ her appreciation, and apologise for the confusion arising from the dense notation, which we will thoroughly revise.

1. *The symbols used in this paper are messy. For example, "1" and "2" are subscripts in Eq.(2) but become superscripts in Figure 5. Besides, there are many symbols not explained such as mj, Hj, Ψ0, etc. Also, I don't know if $x_{j,i}^{(1)}$, $x_{j,i}^{(2)}$ in Figure 5 are same with x_{ij1} and x_{ij2} in Eq.(3). There are many places mismatch, authors should check carefully.*
2. *Why the equations in Fig.5 are totally different with Section 4.2? For example, $pj \sim Beta(\alpha j ,\beta j)$ in Fig.5 but $\rho j \sim Beta[-1,1](d1, d2)$ in Eq.(8).*

We apologise for the notational confusion, this will be fully revised.

3. *The paper involves a lot of hyper-parameters which doesn't demonstrate their selection. For example, c1, c2, d1, d2.*

This is a good point. We will include a sensitivity analysis on the hyperparameters, justifying the choices on both simulated and real data.

4. *In section4.8, I am confused about ρ_j the experiment 2, 5, 8, 11. Why ρ_j both represents ZI rate and correlation?*

We apologise for the notational oversight, which will be rectified.

5. *In Section 4.5, it is difficult to understand the sentence "for me threshold u". Besides, what is r represent in Section 4.5?*

We apologise for the confusing sentence. r is the Pearson correlation coefficient, as explained at the start of 4.5

6. *Why there is "(6a)Agreement between SCRaPL and Pearson" in Fig. 4?*

This simply means that the panel shows a methylation/ expression scatterplot for a gene where estimation by SCRaPL and Pearson return both a significant association. We will expand the caption to explain further.

7. *For Fig.1, I cannot see the text in the rectangle.*

Apologies, we will improve the readability of the figures

8. *I would like to see the efficiency analysis for SCRaPL.*

We have now performed a preliminary analysis using the new Tensorflow implementation, comparing both MCMC and Variational Inference and showing good scalability. The results are summarized in the figure below.

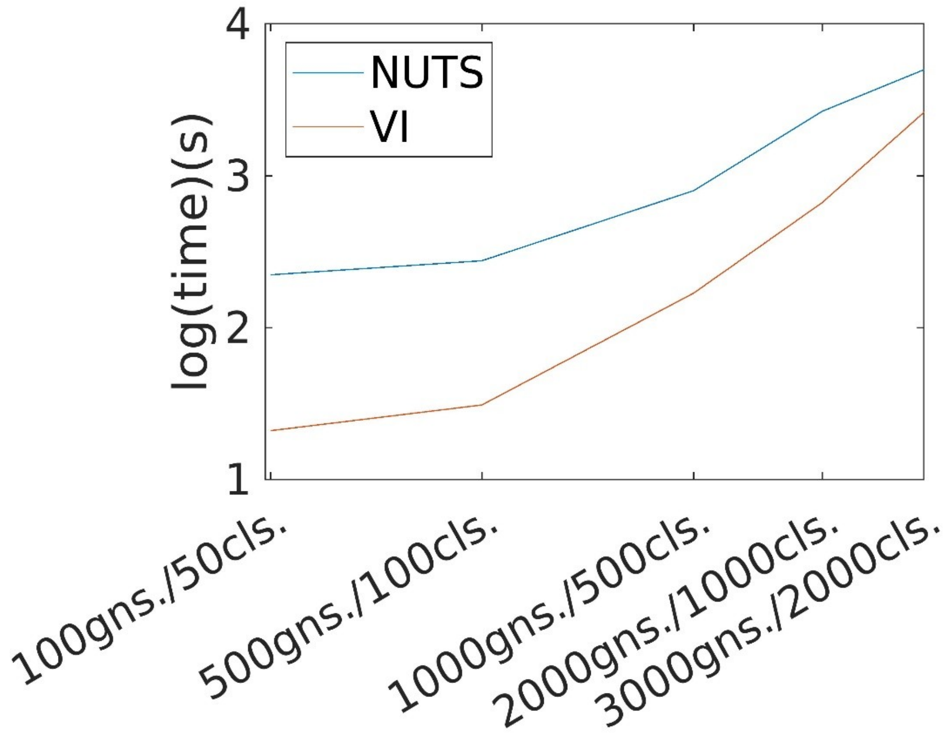

Fig R1: Execution times vs data size (genes/cell numbers)

Reviewer 2

The authors present a Bayesian model to determine noise-corrected correlation coefficients for gene expression (RNA) and DNA-methylation data at single-cell resolution. The authors present a series of simulation data and an example of matched multi-omics data, and compare their results with Pearson correlation. Noise modelling allows the model to determine gene-methylation correlation patterns more accurately. While the authors demonstrate a neat application on accurate quantification of correlation coefficients, I see a limited use of the model for the broader single-cell community. The authors may therefore improve their manuscript on several aspects.

We thank the reviewer for the encouraging words, and thank him/ her for the critical observations, which we have taken at heart, considerably broadening the scope of our paper to make it more attractive to a larger community.

- Abstract: please specify the omics layers that you are analyzing (RNA + DNA methylation) in the abstract

We acknowledge that, while SCRaPL is potentially general, in the first submission we focused only on RNA and DNA methylation. We have now decided to expand our analyses to include 10X data of simultaneous chromatin accessibility (ATAC-seq) and RNA.

- What is the benefit of using a Bayesian model formulation in this setting?

The benefit is twofold: a principled treatment of noise, and a quantification of the resulting uncertainty which allows for a meaningful way to compute Bayesian significance levels. We will expand the discussion of the relative merits of a Bayesian vs frequentist approach.

- Does it also apply to unmatched data?

In principle, given measurements with the same number of cells in all modalities, it is possible to apply SCRaPL. However, unless there is a natural pairing between different cells, the scaling of this approach will be quadratic in the number of cells, hence potentially expensive (although largely parallelizable). We will discuss this now, particularly in the light of applying SCRaPL in conjunction with other suites such as Seurat.

- *Would SCRaPL allow for differential correlation testing?*

At the moment, SCRaPL does not allow for differential correlation testing. Of course, one may run SCRaPL separately on two groups of cells and compare the resulting estimates, which would be informative. Nevertheless, extending SCRaPL to perform differential correlation testing (e.g. using Bayesian model selection) would be a non-trivial effort. We will add a comment on this issue to the discussion section.

- *Figure 1: The graphical description of the model is rudimentary. I believe that the model description could profit from a graphical model representation of SCRaPL (as presented in figure 5).*

We will redraw Fig. 1 and incorporate the graphical model from Fig 5.

- Simulated data: all experiments seem to have rather low cell numbers (max. 200) and genes (max. 300). Given that 10X Genomics is the most widely-used sequencing platform with approx. 10,000 cells and 3,000 (highly variable) genes per experiment, and given that the authors show a use-case with 9480 genes in 487 cells, it seems appropriate to extend the simulations and runtime estimates of the presented model to several thousands of cells and genes, respectively.

Thank you for this comment. The original simulation settings were designed with scMT data in mind, where indeed only a few hundred cells can be assayed at most. Partly because of this feedback, and also because of the request of implementing SCRaPL in a different language, we are now developing a more scalable Tensorflow implementation which could potentially handle thousands of cells and genes in a matter of tens of minutes (see Fig R1 above). The new simulated data will therefore extend into this regime with larger data sets.

- Figure 4: Please revise the figure legend as I did not understand the plotted results based on the description.

We will do so.

- Results section 2.5: Please formulate your whole argument about epigenetic regulators. I do not think that "For further information please refer to supplementary figure XYZ." Is an appropriate closing statement for a paragraph, nor does it motivate the reader to look at the supplementary figures (I did look at them and I do not see how they support the point made in the paragraph). Please elaborate and consider a "take home message" for the paragraph such that the reader is able to understand the benefit of SCRaPL without revisiting the original data publication.

Thank you for this pointer, we will take it on board in the full revision.

- Conclusion: The authors mention that SCRaPL would further offer a "template for the application of more complex analysis techniques (such as clustering, dimensionality reduction and network inference)". If that was the case, the authors should consider a comparison to other tools, which offer exactly that (e.g. Seurat's CCA or non-negative matrix factorization in LIGER). Further, the authors should set their work into context with tools like bindSC.

Thank you for the suggestion. As far as we can tell, all of these methods are thought for unmatched data, rather than multi-omics assays performed in the same cells. Having said that, it is in principle possible to “preprocess” data with SCRaPL and then feed to Seurat or other tools the latent means computed by SCRaPL. We will include an example of how this may be done in the revision.

- Implementation: Matlab is used in about 6% of the single-cell RNAseq tools (according to scrna-tools.org). To reach a larger scientific community, do the authors plan to provide an R or Python implementation of their model?

We have now implemented SCRaPL in Python using Tensorflow probability, achieving substantial speedups (see response to previous point).

Additional minor points about formatting by Reviewer 2 will all be addressed.

Reviewer 3

Maniatis et al propose a sound strategy to analyse single-cell multi-comic data sets. A key advance is to use bespoke error models for each of the omics data. These are integrated into a multivariate gaussian model. This method is a novel and, in my opinion, a valuable addition to the analyses of the growing multi-omics single-cell data sets.

We thank this reviewer for his/ her appreciation of our work.

- Authors make a convincing argument of the importance of principle methods and in particular to use noise models that tailored to the data at hand. To further support this, can authors elaborate on how results would be different from using commonly applied methods ? Eg those embedded in the Seurat, OSCA, and scanpy 'suites'? Authors compare to Pearson correlation-based methods but is not clear if that is the true state-of-the-art on those methods

As far as we know, volcano plots of p-value versus Pearson correlation are the most commonly employed approaches to assess correlations amongst different molecular modalities in single-cell multi-omics (see e.g. Argelaguet et al, Nature 2020). Seurat and other methods normally do not deal with single-cell multi-omics (i.e., multiple omics measured in the same cell), rather with multiple single-cell omics (different molecular modalities assayed in different cells). Nevertheless, it is possible to pre-apply SCRaPL to non-matched data and then use another suite; as an illustration, we will perform an analysis on scMT data using SCRaPL followed by Seurat.

- In the case study on mouse embryonic stem cells, authors excluded the chromatic accessibilty. Why not using it to more clearly show the value of the method?

We did use SCRaPL also on chromatin accessibility, however the signal was weaker and we did not include it in the manuscript, we will now present these results as supplementary material.

- It would also be great if authors would use a different single-cell multi-comic data sets, using other dat modalities, e.g. CITE-Seq data. If this not possible, at least they should elaborate on which omics SCRAPL can handle, what would be the noise models for different data types, etc.

We have started analysing a joint scATAC-scRNA- seq data set generated using the new 10X commercial platform, and will add the results of this analysis to the revised manuscript. We will also expand the description of the suitability for different data types.

- As the authors acknowledge, computational burden is high, which presumably limits scalability. Are authors able to further explore this (scalability on Insilico data)? Or how complex is adopting the variational inference method suggested? I appreciate that the variational inference implementation might be out of the scope of this paper, though.
- It is a pity that the method is in Matlab. Nearly no-one in single-cell omics use

Matlab. Our own lab is largely invested in this topic and we do not even have Matlab licenses. I strongly encourage authors to implement their method in e.g. R or python, ideally compatible with the broadly used 'suites' (Seurat, OSCA, and scanpy,...).

We jointly addressed these two comments by re-implementing SCRaPL in Tensorflow probability (Python based), which also allowed us to leverage powerful libraries for variational inference. This could potentially lead to a substantial increase of scalability, providing the possibility of running on thousands of cells / genes in under one hour (see Fig. R1).
