# Supplementary material for: SCRaPL: A Bayesian hierarchical framework for detecting technical associates in single cell multiomics data
Source: PLoS Comput Biol. 2022 Jun 21;18(6):e1010163. doi: 10.1371/journal.pcbi.1010163 (PMC9249169; doi:10.1371/journal.pcbi.1010163)
Supplement: Attachment — Submitted filename: Response_to_reviewers.pdf [file pcbi.1010163.s014.pdf]

Detailed response to the reviewers' queries

Reviewer 1

This paper proposes a noise-aware approach SCRaPL for modelling the associations of single cell multi-omic data. For gene expression, it uses Poisson-lognormal model. For DNAm data, it uses Binomial noise model which explicitly takes into account the average within the region. The Bayesian hierarchical framework employed by SCRaPL could achieve higher sensitivity and better robustness in identifying correlations, and also offer a template for the application of more complex analysis techniques to multi-omics data. The symbols of this paper are a little bit confusing, and I suggest authors to carefully check them.

We thank the reviewer for his/ her appreciation. In the revised version the notation has been thoroughly revised.

1. *The symbols used in this paper are messy. For example, "1" and "2" are subscripts in Eq.(2) but become superscripts in Figure 5. Besides, there are many symbols not explained such as mj, Hj, Ψ0, etc. Also, I don't know if x_{j,i}^{(1)} , x_{j,i}^{(2)} in Figure 5 are same with x_{ij1} and x_{ij2} in Eq.(3). There are many places mismatch, authors should check carefully.*

2. *Why the equations in Fig.5 are totally different with Section 4.2? For example, pj ~Beta(αj ,βj) in Fig.5 but ρj ~ Beta[−1,1](d1, d2) in Eq.(8).*

We apologise for the confusion, in the revised version the notation has been thoroughly revised.

3. The paper involves a lot of hyper-parameters which doesn't demonstrate their selection. For example, c1, c2, d1, d2.

A sensitivity analysis on correlation prior parameters (d1,d2) can be found in supplementary material S5. Correlation prior parameters were tuned to minimize false positives in negative control data.

4. *In section4.8, I am confused about $ρ_j$ the experiment 2, 5, 8, 11. Why $ρ_j$ both represents ZI rate and correlation?*

The notation oversight has been rectified. As synthetic data experiments were moved in the supplementary materials, the reviewer is advised to look at section S3 .

5. *In Section 4.5, it is difficult to understand the sentence "for me threshold u". Besides, what is r represent in Section 4.5?*

We apologise for the confusing sentence. In relevant section we use r to denote Pearson correlation as explained in the beginning. “for me threshold u” was changed to “for some threshold u” as seen in line 381.

6. *Why there is "(6a)Agreement between SCRaPL and Pearson" in Fig. 4?*

Our intention was to explain that panel 6a showed a methylation/expression scatter-plot for a gene where both SCRaPL and Pearson predicted a significant association. Hopefully, the revised caption below Fig. 4 (now Fig. 3) is easier to understand.

7. *For Fig.1, I cannot see the text in the rectangle.*

We have made the text larger. Hopefully the figure is more readable.

8. *I would like to see the efficiency analysis for SCRaPL.*

Efficiency analysis can be found in supplementary materials section S10. In our preliminary analysis we experimented with both No -U – Turn sampler and Variational inference. Unfortunately Variational inference performance was not up to required standards and was abandoned. As we can see in figure S20 for large problems SCRaPL scales linearly in genes and cells.

Reviewer 2

The authors present a Bayesian model to determine noise-corrected correlation coefficients for gene expression (RNA) and DNA-methylation data at single-cell resolution. The authors present a series of simulation data and an example of matched multi-omics data, and compare their results with Pearson correlation. Noise modelling allows the model to determine gene-methylation correlation patterns more accurately. While the authors demonstrate a neat application on accurate quantification of correlation coefficients, I see a limited use of the model for the broader single-cell community. The authors may therefore improve their manuscript on several aspects.

We thank the reviewer for the encouraging words, and thank him/ her for the critical observations, which we have taken at heart, considerably broadening the scope of our paper to make it more attractive to a larger community.

- Abstract: please specify the omics layers that you are analyzing (RNA + DNA methylation) in the abstract

We acknowledge that, while SCRaPL is potentially general, in the first submission we focused only on RNA and DNA methylation. We have now decided to expand our analyses to include 10X data of simultaneous chromatin accessibility (ATAC-seq) and RNA. More information about the data we used can be found in section “SCRaPL improves the power to identify associations between molecular layers in mouse embryonic stem and brain cells” (line 91).

- What is the benefit of using a Bayesian model formulation in this setting?

The benefit is twofold. To begin with, the principled treatment of noise allows us to account for coverage and sparsity, making SCRaPL more robust to outliers and better at uncovering biological signal. For more information look at sections " SCRaPL associations are influenced by data sparsity and are robust to outliers." (line 140) and "SCRaPL identifies biologically meaningful epigenetic regulation in early mouse gastrulation " (line 183). Then quantification of uncertainty allows for a meaningful way to compute Bayesian significance levels. As a result, we observe 3-5 times more significant features compared to Pearson (see lines 110-125), with many of the important features found by Pearson also identified by SCRaPL (see figure2) and a low number of false positives (see supplementary tables S1-S3).

- Does it also apply to unmatched data?

In principle, given measurements with the same number of cells in all modalities, it is possible to apply SCRaPL. However, unless there is a natural pairing between different cells, the scaling of this approach will be quadratic in the number of cells, hence potentially expensive (although largely parallelizable). We will discuss this now, particularly in the light of applying SCRaPL in conjunction with other suites such as Seurat.

- *Would SCRaPL allow for differential correlation testing?*

At the moment, SCRaPL does not allow for differential correlation testing. Of course, one may run SCRaPL separately on two groups of cells and compare the resulting estimates, which would be informative. Nevertheless, extending SCRaPL to perform differential correlation testing (e.g. using Bayesian model selection) would be a non-trivial effort. We will add a comment on this issue to the discussion section.

- Figure 1: The graphical description of the model is rudimentary. I believe that the model description could profit from a graphical model representation of SCRaPL (as presented in figure 5).

We have revised figure 1.

- Simulated data: all experiments seem to have rather low cell numbers (max. 200) and genes (max. 300). Given that 10X Genomics is the most widely-used sequencing platform with approx. 10,000 cells and 3,000 (highly variable) genes per experiment, and given that the authors show a use-case with 9480 genes in 487 cells, it seems appropriate to extend the simulations and runtime estimates of the presented model to several thousands of cells and genes, respectively.

Thank you for this comment. The original simulation settings were designed with scMT data in mind, where indeed only a few hundred cells can be assayed at most. In the revised version we have taken steps to address that. In experiments with synthetic data located in section S3 of supplementary materials we have extended the simulations up to 1600 cells. Above that level, attempts to break the model using data from different observation generating distributions were not successful. Synthetic data with more than 300 genes did not yield significantly different plots from the ones in supplementary figures S2-S10 due to independence assumption among genes. We did run some

experiments for synthetic data ranging from 100 genes and 50 cells to 4000 genes and 4000 cells to estimate complexity. Results did not change compared to plots in figures S2-S10. Runtime estimates can be found in supplementary figure S20.

- Figure 4: Please revise the figure legend as I did not understand the plotted results based on the description.

As explained above, our intention was to indicate different scenarios where SCRaPL and Pearson agree and disagree on their predictions. Hopefully revised caption below Fig. 4 (now Fig. 3) is easier to understand.

- Results section 2.5: Please formulate your whole argument about epigenetic regulators. I do not think that "For further information please refer to supplementary figure XYZ." Is an appropriate closing statement for a paragraph, nor does it motivate the reader to look at the supplementary figures (I did look at them and I do not see how they support the point made in the paragraph). Please elaborate and consider a "take home message" for the paragraph such that the reader is able to understand the benefit of SCRaPL without revisiting the original data publication.

Thank you for this pointer, we have amended the entire section 2.5 (now under the title "SCRaPL identifies biologically meaningful epigenetic regulation in early mouse gastrulation" (line 183). The main message of this section and in general of this work is that technical variability erodes correlation and under-powers exploratory data analysis. Hence by taking into account sources of variation we can identify genomic regions with known strong regulatory behavior, but more importantly we might point to less known ones. This can be found in lines 207-211

- Conclusion: The authors mention that SCRaPL would further offer a "template for the application of more complex analysis techniques (such as clustering, dimensionality reduction and network inference)". If that was the case, the authors should consider a comparison to other tools, which offer exactly that (e.g. Seurat's CCA or non-negative matrix factorization in LIGER). Further, the authors should set their work into context with tools like bindSC.

Thank you for the suggestion. As far as we can tell, all of these methods are thought for unmatched data, rather than multi-omics assays performed in the same cells. Having said that, it is possible to "preprocess" data with SCRaPL and then feed to Seurat or other tools the latent means computed by SCRaPL. This analysis can be found in section "Using SCRaPL as a data denoising tool" (line 212)

- Implementation: Matlab is used in about 6% of the single-cell RNAseq tools (according to scrna-tools.org). To reach a larger scientific community, do the authors plan to provide an R or Python implementation of their model?

We have now implemented SCRaPL in Python using Tensorflow probability, achieving substantial speedups.

Additional minor points about formatting by Reviewer 2 have been addressed.

Reviewer 3

Maniatis et al propose a sound strategy to analyse single-cell multi-comic data sets. A key advance is to use bespoke error models for each of the omics data. These are integrated into a multivariate gaussian model. This method is a novel and, in my opinion, a valuable addition to the analyses of the growing multi-omics single-cell data sets.

We thank this reviewer for his/ her appreciation of our work.

- Authors make a convincing argument of the importance of principle methods and in particular to use noise models that tailored to the data at hand. To further support this, can authors elaborate on how results would be different from using commonly applied methods ? Eg those embedded in the Seurat, OSCA, and scanpy 'suites'? Authors compare to Pearson correlation-based methods but is not clear if that is the true state-of-the-art on those methods

As far as we know, volcano plots of p-value versus Pearson correlation are the most commonly employed approaches to assess correlations among different molecular modalities in single-cell multi-omics (see e.g. Argelaguet et al, Nature 2020). Seurat and other methods normally do not deal with single-cell multi-omics (i.e., multiple omics measured in the same cell), rather with multiple single-cell omics (different molecular modalities assayed in different cells). Nevertheless, it is possible to pre-apply SCRaPL to non-matched data and then use another suite; This analysis can be found in section "Using SCRaPL for data integration" (line 212)

- In the case study on mouse embryonic stem cells, authors excluded the chromatin accessibilty. Why not using it to more clearly show the value of the method?

We did use SCRaPL on chromatin accessibility, however the signal was weaker and we did not include it in the manuscript. In the revised the table with detection rates can be found in supplementary table S3.

- It would also be great if authors would use a different single-cell multi-comic data sets, using other dat modalities, e.g. CITE-Seq data. If this not possible, at least they should elaborate on which omics SCRAPL can handle, what would be the noise models for different data types, etc.

We have analysed a joint scATAC-scRNA-seq dataset generated using the new 10X commercial platform, more information can be found in results section. More precisely in lines 96-105 and 405-406 the reviewer will find a high level description of the brain cell data and how they were pre-processed. Moreover, in lines 110 – 125, panels (c) and (d) of figure 2 we have included included a comparison between SCRaPL and Pearson predictions. In lines 316-323 and figure 5b we provide a brief description of the chromatin accessibility noise model and a model comparison for noise model with and without inflation respectively. Then an extensive comparison between SCRaPL and Pearson on specific genomic regions could be found in supplementary materials section S6. As previously mentioned, SCRaPL can handle multiple omics measured in the same cell with the appropriate error model. Therefore, it should be possible to extend our framework to arbitrary types of single cell multi-omics data, including CITE-seq.

- As the authors acknowledge, computational burden is high, which presumably limits scalability. Are authors able to further explore this (scalability on Insilico data)? Or how complex is adopting the variational inference method suggested? I appreciate that the variational inference implementation might be out of the scope of this paper, though.

- It is a pity that the method is in Matlab. Nearly no-one in single-cell omics use Matlab. Our own lab is largely invested in this topic and we do not even have Matlab licenses. I strongly encourage authors to implement their method in e.g. R or python, ideally compatible with the broadly used 'suites' (Seurat, OSCA, and scanpy,...).

We jointly addressed these two comments by re-implementing SCRaPL in Tensorflow probability (Python based), which allowed us to leverage powerful inference libraries. Unfortunately Variational Inference was not as accurate as we would like, so we resorted to NUTS. Our efficiency analysis on section S10 of supplementary material shows that for large problems SCRaPL scales linearly (figure s20). For a dataset with 4000 genomics regions and 4000 cells (~32 million parameters to be inferred), it would take SCRaPL less than 9 hours using a GPU to gather 2000 samples for each parameter after a burn-in of 3000 samples.
