# Supplementary material for: SCRaPL: A Bayesian hierarchical framework for detecting technical associates in single cell multiomics data
Source: PLoS Comput Biol. 2022 Jun 21;18(6):e1010163. doi: 10.1371/journal.pcbi.1010163 (PMC9249169; doi:10.1371/journal.pcbi.1010163)
Supplement: Attachment — Submitted filename: Response to reviewers.pdf [file pcbi.1010163.s015.pdf]

Dear Editor,

we are writing to you to resubmit a revised version of our manuscript "SCRaPL: hierarchical Bayesian modelling of associations in single cell multi-omics data". We thank you and the reviewers for the prompt and thorough reviewing of our manuscript, and for the constructive feedback offered by the reviewers. We thoroughly revised the paper in the light of their comments, and believe it is now ready to be published.

A detailed point-by-point response to every issue raised by the reviewers is provided in the appended document. Briefly, the major changes we enacted are the following:

- we introduced an additional comparison to Spearman's rank correlation statistic, providing further insights into the applicability domain of the various methods considered.
- we incorporated part of the simulated data analysis in the main text, to set the stage for the main discussion on real data.
- we revisited the presentation and revised the notation to make the paper more clear and legible.

We trust that with these changes the manuscript will be considered acceptable, and look forward to hearing from you soon.

Best regards,

Christos Maniatis, Catalina Vallejos and Guido Sanguinetti

Description of revisions

Reviewer 1:

In this work, the authors developed a method to identify region-gene association based on single-cell multi-omics data. The method is based on a Bayesian hierarchical model, which uses zero-inflated Poisson model with logit link function for the modalities of gene expression and chromatin accessibility, and binomial model with probit link for the modality of methylation. Overall, the method and study look solid. This is also an important problem in single-cell multi-omics. This could be a nice addition to the literature. I think Spearman correlation is more commonly used under this setting, because of its robustness to outliers. It will be good to also include Spearman correlation in the comparison.

We thank the reviewer for his/ her appreciation. We have now included Spearman as a comparator method, both on synthetic and real data. Our results show that, for high-coverage data (like the one from plate-based technologies), Spearman performs very similarly to Pearson.

Reviewer 2:

Maniatis et al proposes a methods named SCRaPL to investigate the correlation in multi-omics single-cell datasets. The method is novel in its usage of a Bayesian hierarchical model to infer

associations between different omics components. If the claim that the method has higher power and a good control on false positives can be better supported in analysis, this will be a potentially useful method in single-cell studies.

We thank the reviewer for his/her feedback.

Writing:

Since a significant amount of descriptions is included in the supplementary file, the authors need to improve the clarity of the main text to help readers navigate between the manuscript and the supp file. I found myself spending a lot of time searching for explanations in the supp file.

We have now clarified the model description and tidied up the notation. We have also moved to the main text some of the main analyses on simulated data, thus reducing the need for the reader to consult the supplementary file.

Methods:

It is not clear to me what's the meaning of Y. From formula (3), it seems that it should represent raw counts. However, the supplementary methods mention that the RNA data is normalized during preprocessing. Please clarify.

Indeed, we use Y{ij1} for raw expression count. The text has been adapted to reflect that. Our normalization strategy is to estimate a cell specific normalization constant using scran which in turn we use as a model offset. The only place we normalize expression by dividing with normalization constant is when we estimate Pearson/Spearman correlation.

The authors need to explain how the data (except for gene expression) is binarized in order to use the Binomial distribution. Would the binarization cutoff significantly impact the final results?

We have now edited Supplementary Section S1 to clarify this. We note that binarization is only applied at the level of individual CpGs. For example, as in BPRmeth (https://doi.org/10.1093/bioinformatics/btw432), when analyzing DNA methylation data, CpGs are labeled as methylated (>50% methylation rate) or not. The input data for SCRaPL is obtained by aggregating these binary events at the region level (e.g. promoter) in terms of the number of methylated CpGs among those for which the methylation status has been measured.

Is there any justification for the usage of the probit link function in formula (4)?

The justification for the use of a probit link is primarily computational, as it yield a tractable Gibbs sampler update (which was used in the prototyping of the model and still is implemented in the MATLAB version of the model). Conditional Binomial /Bernoulli error model with probit link have been used previously in various methods, also for single-cell epigenomic studies (ie. https://doi.org/10.1093/bioinformatics/btw432, https://doi.org/10.1186/s13059-019-1665-8).

Key derivation steps to obtain the posterior distribution are not given. The distribution should be added to Method and the key steps should be included at least in the supp file.

SCRaPL's posterior distribution is not tractable, so it is impossible to obtain a closed form posterior. For inference we input the full graphical model to TensorFlow probability which then efficiently runs

NUTS (a state of the art variation of Hamiltonian Monte Carlo) to draw samples from the posterior distribution.

No software package is available for others to use the method.

SCRaPL's code can be found in https://github.com/chrmaniatis/SCRaPL. At the moment there is no package but all experiments could be reproduced from jupyter notebooks. We acknowledge the inconvenience this might cause, but we currently do not have the resources to turn SCRaPL into an easily deployable package.

Results:
In the experiments with synthetic data,
(1) what's the definition of "gene coverage"?

Gene coverage refers to the total number of CpG's/GpC's WGBS captures. For more information look at binarization cutoff question above.

(2) I would suggest moving the plots of true and inferred correlations to the main manuscript.
We agree that presenting results on synthetic data at the beginning of the results section in the main text improves the overall flow of the paper. We now present excerpts of the synthetic data results in the "Benchmarking SCRaPL using synthetic data' section, including a modified plot on true/ inferred correlations as the number of cells varies (with a direct comparison with Spearman and Pearson) and a scatterplot of inferred correlations in Figure 2.

(3) The Method section describes the approach to identify statistically significant correlation using SCRaPL. Can the authors show the accuracy of this method on these datasets?

Pearson correlation is currently used to assess epigenetic regulation in different genomic regions. With SCRaPL we are trying to demonstrate the pitfalls of using a tool that does not take into account the types of noise present in different multi-omics layers. Unfortunately, there is no ground truth to estimate accuracy metrics outside in silico setting (Supplementary figures S2e,S3e,S4e). Hence, we resort to ad-hoc approaches like negative control experiments found in supplementary S4. In these experiments we permute cells to remove correlation from real data and demonstrate that under such circumstances the number of false positives stays below 10%. So, the take home message is that with proper noise treatment one can detect significantly more features compared to Pearson/Spearman while being sure that in cases of correlation absence it is unlikely for the model to produce a false positive.

In the analysis of mESC data, "a dataset with 9480 features and 679 cells" was used. This number is much smaller than the possible number of features. How many genes or DNAm features are included in these 9480 features? How would it affect the performance of SCRaPL if a less stringent filtering is applied and more features are included? Similar questions apply to the mEBC data.

In this paper, features is an umbrella term for different genomic regions. More precisely in case of mESC data features mean gene promoters ±2.5kbp around Transcription Start Site (TSS). In case of mEBC data, we link enhancers to genes at most 12.5kbp away. The resulting number of retained features is aligned with the standard of many single-cell 'omics papers, because the high sparsity of the data causes normally a substantial fraction of genes/ cells to be discarded. It should be stressed

the mESC data set uses the plate-based scNMT technology, which assays a smaller number of cells than 10x technologies (with the benefit of greater coverage though). Results are not significantly affected by changes in the filtering parameters within reasonable range (as demonstrated by the synthetic data analysis where we vary such parameters).

Can the authors also show the comparison between SCRaPL and Pearson's correlation (power and false positive rate) using the synthetic data?

We now show such results in Figure 2.

The last Results section presents SCRaPL as a data denoising method, and performs Seurat integration with and without SCRaPL's preprocessing.
(1) From Figure 4, it is not clear to me that SCRaPL's preprocessing improves the analysis. Can the authors provide some quantitative comparisons?
Indeed, the reviewer is correct to point out that SCRaPL does not offer important improvements in Seurat's integration pipeline. Our analysis was a proof of concept to explore the extent to which the denoising component of a hierarchical model, designed to address weaknesses offered by standard tools, can offer any improvement to CCA. As it is also noted in the text, the improvement (if any) is minuscule as "CCA components do an excellent job at filtering our noise."

(2) A more detailed description needs to be provided in Methods. With SCRaPL's preprocessing, what data is provided as the input into Seurat?

As noted in sections "Using SCRaPL as a data denoising tool" and "Single cell multi-omics datasets" SCRaPL is used to process 3000 PBMCs (downsampled from 12000) from (https://support.10xgenomics.com/single-cell-multiome-atac-gex/datasets/1.0.0/pbmc_granulocyte_sorted_10k). We used SCRaPL to estimate noise-free latent accessibility/expression space of 60000 features investigating to what extent it can improve CCA used in integration. Changes are marked in blue and can be found under section "Using SCRaPL as a data denoising tool".
(3) Since the procedure involves sampling from posterior distributions, how different are the integration results if the data are sampled multiple times?
We have performed stability analysis by using three posterior latent space samples. Relevant changes can be found in Supplementary section S12, supplementary figure s24 and under the section "Using SCRaPL as a data denoising tool"

Reviewer 3:
It seems that the author has largely addressed previous reviewers' comments. However, the authors need to check if every single comment has been replied. For example I don't see response for the first comment of the first reviewer. Also, the figure legends need to be improved to discuss each of the subplots. Such description is lacking for figures 2 and 4.

We thank the reviewer for his/her time to review our work. We did not specifically address comments related to typos/ syntax errors in our response but we incorporated all such comments brought to our attention.

For the software package on GitHub, I don't see any instructions about how to use the software or how to reproduce the results in the paper. This needs to be significantly improved.

We apologize for the inconvenience. The revised repository includes demos on how to run SCRaPL on data both from droplet and plate based technologies. Furthermore, we have included readme files that help users to navigate through the repository.
